# InChI, the IUPAC International Chemical Identifier

**DOI:** 10.1186/s13321-015-0068-4

**Published:** 2015-05-30

**Authors:** Stephen R Heller, Alan McNaught, Igor Pletnev, Stephen Stein, Dmitrii Tchekhovskoi

**Affiliations:** Biomolecular Measurement Division, National Institute of Standards and Technology, Gaithersburg, MD 20899-8362 USA; InChI Trust, Cambridge, UK; Department of Chemistry, Lomonosov Moscow State University, 119991 Moscow, Russia

**Keywords:** InChI, InChIKey, Chemical structure linear notation, Chemical identifier, IUPAC standard

## Abstract

This paper documents the design, layout and algorithms of the IUPAC International Chemical Identifier, InChI.

## Introduction

InChI is the International Chemical Identifier developed under the auspices of IUPAC, the International Union of Pure and Applied Chemistry [1], with principal contributions from NIST (the U.S. National Institute of Standards and Technology [2]) and the InChI Trust [3].

This paper documents the design, layout and algorithms of InChI. It is intended to provide a reasonably detailed description without being overlong for a journal article. For a briefer introduction, which also provides more detail on historical and organizational matters, the reader is referred to a recent paper by the same authors [4]. For a more technical description, one may consult the InChI Technical Manual [5] and the free source codes of the InChI software, which are available from the InChI Trust [6].

The paper is organized as follows. First, we discuss the general concepts associated with chemical identifiers. Then we outline the design goals of InChI and our general approach, focussing on the InChI model of chemical structure and the hierarchical layered structure of the Identifier; the concept of Standard InChI is introduced. This is followed by a detailed description of each of the possible major InChI layers, accounting for molecular connectivity, charge, stereochemistry, isotopic enrichment, position of hydrogen atoms and bonding in metal compounds, and the sublayers associated with these layers. We then describe the workflow of InChI generation (normalization, canonicalization, and serialization stages), as well as generation of the compact hashed code derived from InChI (InChIKey); the related algorithms and implementation details are briefly discussed. Finally, we provide information about InChI Software, licensing, known problems/limitations, and future prospects for InChI.

## Background

A chemical identifier is a text label that denotes a chemical substance^a^. These labels are of the utmost importance as they provide a convenient means of comparing and distinguishing chemicals in a variety of applications, from the design of new materials to legal and regulatory issues.

The main requirement for an identifier is that the label must be unambiguous: the same label must always refer to the same substance, and no other substance may have this label. Two different substances must have different labels.

Note that an identifier may not be strictly unique in the sense that the same substance may be, on a case-by-case basis, denoted by several distinct synonymical labels (provided that the lists of synonyms for different substances do not overlap). An obvious example is given by IUPAC chemical nomenclature that allows one to produce and use different names for a single compound; nevertheless, all these names unambigously identify the compound. Though not necessary, strict uniqueness, which is always assigning a single label to a particular substance, is highly convenient and very desirable.

The concept of “chemical identifier” heavily relies on the concepts of chemical substance and chemical identity. The IUPAC Compendium of Chemical Terminology, the “Gold Book”, defines “chemical substance” as “Matter of constant composition best characterized by the entities (molecules, formula units, atoms) it is composed of. Physical properties such as density, refractive index, electric conductivity, melting point etc. characterize the chemical substance” [7]. As this definition implies, identity of a chemical substance is determined by its constituent units and properties. It is noteworthy that even at this highly general level, this consideration is somewhat restrictive. For example, the concept of “chemical substance” is not applicable to material that is not of constant composition (e.g., oil). This consideration is also somewhat counter-intuitive, for example, as concerns aggregate states, polymorphs, etc. Thus, most chemists would agree that “water” is a chemical substance, that may appear as steam, ice and liquid water, and that all three should have the same chemical identifier -- despite the fact that each may be isolated as a different state of matter, placed in a test tube or stored in a bottle. In other words, the “identifying power” of a chemical identifier is inherently limited.

The oldest known chemical identifiers are words of natural languages describing common chemicals with terms like “water”, “iron” or “table salt”; they are *trivial names*, in modern nomenclature. Notably, trivial names exemplify the principle that a chemical identifier is not necessarily related to molecular structure. The identity of chemical substances denoted by trivial names was historically determined by a set of characteristic physical and chemical properties, long before exact structures were resolved, or even before the very concept of molecular structure evolved in the second half of the 19th century. Of course, today’s trivial names are associated with chemical stuctures (yet the structures may not be fully defined, as is common for natural products).

A trivial name is an example of a *registry-lookup* chemical identifier: it provides a unique label for the named substance but the label itself says nothing (or little) about the characteristic properties and structure. Such data are stored in electronic or printed registries (handbooks) that uniquely associate the label with the properties/structure. Retrieving reference data requires a registry lookup.

More recent examples of registry-lookup identifiers are those associated with large printed or electronic collections of chemical structures and properties – Beilstein and Gmelin Registry numbers [8], Chemical Abstracts Service (CAS) Registry numbers by the American Chemical Society [9], EC numbers from the European Community Inventory [10], CID and SID numbers assigned by PubChem [11], and identifiers assigned by ChEMBL [12], ChemSpider [13], etc.

Note that all the above registry-lookup identifiers are also *authority-assigned* identifiers, that is, they are produced by assignment made by some authority. Typically, the authorities are the maintainers of chemical substance collections e.g. CAS numbers are assigned by Chemical Abstracts Service, and one needs to refer to CAS for a particular structure’s identifier.

(Trivial chemical names provide an interesting exception: these were/are assigned by the chemical community and their associated registry is a decentralized compendium of handbooks and nomenclature rules. In these cases, there is no algorithm for direct conversion of molecular structures to these identifying labels).

Despite the widespread use of *registry-lookup authority-assigned* chemical identifiers, these types of identifier have a number of substantial drawbacks. For example, even the largest registry cannot include all known chemical substances. Furthermore, no registry can include a substance that has not previously existed and for which a hypothesized structure is drawn or computed. Furthermore, some authorities may impose restricted access and/or require payment for assigning labels and even for lookup in their registries.

The alternative to *authority-assigned* is *structure-based* chemical identifiers. These are derived from molecular structural formulae, either drawn in print form or presented in digital form. When the algorithm for an identifier’s derivation and/or a related utility tool becomes publicly available, anyone has the ability to produce the identifier for a given structure. (Note that structure-based identifiers still may require registry lookup to recover the structure from the label).

**Figure 1:**
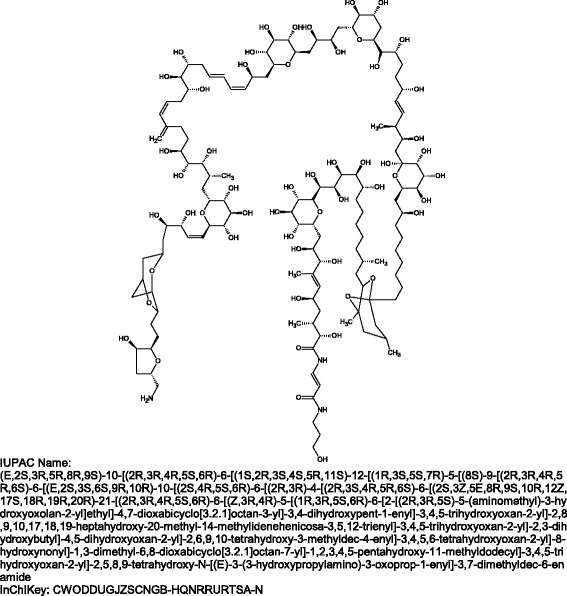
Structure, IUPAC name and InChIKey for palytoxin [14].

The earliest examples of structure-based identifiers are the systematic names of classical chemical nomenclatures established either by IUPAC or by CAS. However, the nomenclature rules developed by these authorities are not easy to learn and practice, even for professional chemists. Misinterpretation may result in ambiguous naming. Finally, and most importantly, systematic chemical names are not well suited for digital representations and the internet: they tend to be too long and contain non-alphanumeric characters (i.e., other than Latin letters and digits). As an example, Figure 1 gives the IUPAC systematic chemical name for the marine toxin palytoxin [14]; this is compared with the much shorter InChIKey (discussed later).

Since the second half of the 20th century, chemical structure *linear notations* have become well established as alternative to classical nomenclature. These textual labels are derived using specific algorithms from molecular structural formulae. They serve as handy textual substitutes for structural formulae, being much more convenient in database and internet applications. Examples are the pioneering Wiswesser line-formula notation, WLN [15]; the widely used SMILES [16,17]; notation by Sybyl, SLN [18,19]. An excellent review of these and other notations is given in [20].

Typically, these systems encode the chemical structure, which is originally expressed (drawn or computer stored) in the paradigm of classical chemical structure theory [20]; most typically, the source representation is provided in a file using a connection table (CTFile) format (e.g. MOL and SDF, from MDL [21]).

This “classical model of chemical structure” assumes that a molecule is composed of atoms that are connected by bonds. Atoms are characterized by their chemical element, isotopic mass, integer formal charge, radical state and connection to other atoms. It is assumed that elements (more strictly, atoms of a particular element in a particular charge/radical state) have typical *valence states*, characterized by the number of bonds to neighbors. Typically, if the explicitly expressed number of connections is less than the characteristic valence, the necessary number of connections to implicit (not shown) hydrogen atoms are assumed. Atoms do have coordinates, but typically they are *x,y*-coordinates for visually pleasant drawings of structural formulae, which have no relation to *x,y,z*-coordinates of atomic nuclei in physical space. However, these coordinates may be useful to represent stereo configurations of double bonds, as well as other stereogenic elements. Bonds may be of single or multiple order. In some cases, “resonance” or “aromatic” bonds (of “one and a half” order) are also included. In all cases, bonds are pair-wise; no bond may involve three or more atoms.

This model is quite different from the modern quantum chemistry description. In spite of this, it performs surprisingly well in rationalizing chemical facts, and forms a solid basis (mathematically, it is an undirected multigraph with colored nodes) for nearly all structure-based chemical identifiers. One, however, should be cautious, bearing in mind the limitations of the model.

A *line notation* structure-based identifier is, essentially, the connection table (with associated additional data) unfolded to a single line. This unfolding requires the use of a pre-defined order of numbering of atoms in the molecule. In addition, atoms always may be renumbered, and renumbering may (more often than not) change the identifier. In other words, uniqueness of an identifier requires a method for assigning unique, *canonical*, numbers to the atoms.

Unfortunately, although the formats of most of the above mentioned line notations are publicly available (though not necessarily as detailed and formal as desirable), the related algorithms and software are not always available. Even in the cases where the algorithms are described, as is the case for the most widely employed system, SMILES [16,17], the original implementation and software for the algorithms remain proprietary. Moreover, for SMILES, the canonicalization algorithm was published over 25 years ago, but incomplete (without stereochemistry-related part). To compensate for this, “other commercial and open-source software developed their own algorithms for generating canonical SMILES all of which differed from each other and none of which are published” (O’Boyle [22]); however, the lack of the single commonly-adopted standard became a problem itself.

Another problem is that most of the identifiers perceive the structural formulae "just as drawn". This means, for example, that mesomeric structures, which undoubtedly represent the same substance, may surprisingly produce different labels. Also, the tautomers, which are most often presumed to be associated with the same substance (unless otherwise explicitly intended), are often labeled with different identifiers. Stereo isomers and isotopically enriched forms of the same parent compound present an additional source of ambiguity and inconsistent labelling. These factors lead to the undesirable and widespread result where the same substance has different labels, and *vice versa*. This further results in simultaneous problems in cross-referring various forms of the same or "nearly the same" substance (tautomers, stereo isomers, etc.). A typical pattern of cheminformatics work includes a) correction of drawing issues by normalizing to a preferred state and b) re-drawing molecules in intentionally different ways, dependent on context (e.g., including or omitting stereo wedges). The lack of universally-recognized standards for these correction and re-drawing transformations results in a drastic decrease in interoperability.

To address the lack of a non-proprietary, strictly-unique standard chemical identifier, the InChI project was initiated in 2000 by two authorities well-known for establishing standards, IUPAC and NIST.

Since the InChI project was established, four major InChI software releases have appeared and each has introduced significant new features. The history of the development of InChI is documented in earlier reports and accounts [23-27]. For a general overview, we refer the reader to a recent paper [4].

## Design and layout

This section provides information on InChI design goals and the general approach chosen to meet them -- a method of constructing the Identifier which reflects the various features of chemical structure in a hierarchical, layered manner. The concept of Standard InChI, which is specifically designed for inter-operability by selecting the most appropriate layers, is introduced. Then the major InChI layers: Main, Charge, Stereo, Isotopic, FixedH as well as the Reconnected layer, and their associated sublayers, are described in detail.

### InChI design goals

InChI is a non-proprietary, Open Source, chemical identifier intended to be an IUPAC approved and endorsed structure standard representation. The following features were considered as critically important in designing the International Chemical Identifier^b^.

- ▪ Structure-based approach.
  Anybody anywhere should be able to produce InChI from just the structural formula of a chemical substance.

- ▪ Strict uniqueness of identifier.
  The same label always means the same substance, and the same substance always receives the same label (under the same labelling conditions). This is achieved through a well-defined procedure of obtaining canonical numbering of atoms.

- ▪ Non-proprietary, Open Source, free and open approach.
  - o Free access to developed computer programs.
    No payment is assumed under any circumstances.
  - o Open access to the source code.
    Everybody is free to read and use the source code.

- ▪ Applicability to the entire domain of “classic organic chemistry” and, to a significant extent, to inorganic compounds, bearing in mind the eventual goal to extend InChI to cover all of chemistry.
- ▪ Ability to generate the same InChI for structures drawn under (reasonably) different styles and conventions, specifically those represented by mesomers.
- ▪ Hierarchical approach allowing encoding of molecular structure with different levels of “granularity”, dependent on algorithms and software switches. In particular, the ability to include/exclude stereochemical, isotopic and tautomeric information was considered necessary.
- ▪ Ability to produce an identifier with some “default” switches, targeted to a fixed level of granularity and ensuring interoperability in large databases.

The current InChI (InChI identifier version 1, InChI software version 1.04) implements these features in full. Normalization may modify input chemical structure by applying a consistent chemical model with the intent to make structures of the same compound drawn under (reasonably) different styles and conventions close if not identical, which is essential for generating the same InChI. Canonicalization of chemical structure upon generating InChI ensures strict uniqueness of the identifier. The layered structure of InChI allows targeting for specific applications (e.g., adding the ability to distinguish tautomers). A Standard InChI is specifically created for inter-operability. All of the development occurred under open-source paradigm.

The following features were considered important but not critical.

- ▪ Ability to exactly restore the original chemical structure based solely on the InChI identifier string.
- ▪ Compact form.
- ▪ Ability to deal with coordination and organometallic compounds, including those containing haptic bonds.

The current implementation preserves these features to a significant extent. As measured by extent of correct InChI- > Structure- > InChI conversion of a ~39 million structures collection derived from PubChem Compound, the current software correctly restores the structure in ~99.95% cases^c^.

As a response to a requirement for a more compact identifier, a shorter hash-based InChI derivative, denoted InChIKey, was introduced. This came about from a discussion with a search engine company who explained that without a shortened compact version of InChI, no search engine would be able to properly search for a lengthy InChI string.

Organometallics, inorganics and other classes of compounds still present a significant challenge. This will be addressed in future versions of InChI.

The following was considered as having low importance:

- ▪ Ability to be human read/parsed and manually edited.

#### InChI model of chemical structure

InChI is based on the “classical model of chemical structure” with some significant modifications and additions.

The following principles constitute the basis of the InChI approach, or the “InChI model of chemical structure”.

A molecule is composed of atoms. Atoms are either skeletal (non-hydrogen atoms, as well as bridging hydrogen, as in diborane) or terminal hydrogen atoms (further called simply “hydrogens”). Skeletal atoms are pair-wise connected by bonds and are characterized by chemical element, integer formal charge, radical state, isotopic mass, associated implicit hydrogens, and bonds to other skeletal atoms. Hydrogens may be either connected to skeletal atoms or shared by a group of skeletal atoms (such groups may also share negative charge).

All bonds are simple links (connections). That is, they have no "double", "triple" or other attributes. Bonds are formed pair-wise; thus, no bond may involve three or more atoms [except for hydrogen(s) shared by a group of skeletal atoms].

A molecule is coordinateless. However, the identifier represents the configuration of stereogenic elements, as it is captured from source structural data amplified with either 2-D or 3-D coordinates.

#### Core parent structure

The most important aspect of InChI is its hierarchical, layered nature. At the center of the InChI approach is the concept of *core parent structure*, which is a common archetype for the source structure and many related structures - (a) tautomeric, (b) stereo isomeric, (c) isotopically substituted, and (d) protonated/deprotonated forms. Additionally, (e) all the bonds to metal atoms are broken (although the bonding pattern is saved). The core parent structure has no precise tautomeric state, tautomeric "mobile" hydrogens are assigned to groups of skeletal atoms; it has no associated stereochemistry and no isotopic enrichment. Its protolytic centers are neutral, as the core parent is derived from the source structure by adding/removing the appropriate number of protons (to be more precise, the structure as a whole is neutralized).

InChI describes the source structure as the derivative of its parent core with explicitly added features (items a-e above). The exact description requires specifying all of the five items, if they are applicable. Any other (incomplete) combination may be used. InChIs that have been generated with tautomerism excluded will be the same for the source structure and all of its (recognized) tautomers. Omitting stereo configurations means one will produce the same InChI for the source structure, as well as for all of its stereo isomers, etc.

**Figure 2:**
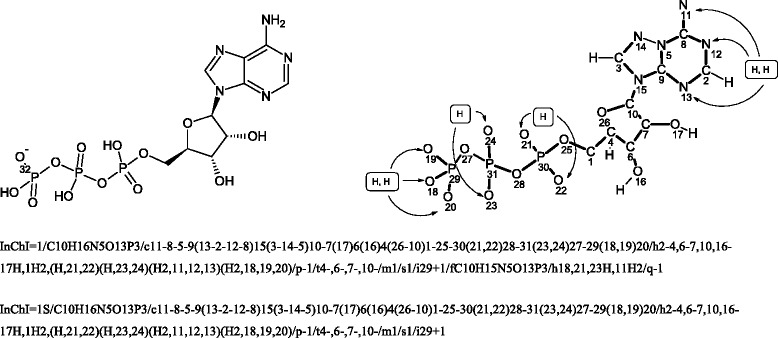
InChI layered representation of the monoanion of ^32^P-labelled adenosine triphosphate. Left – input structure; right –*core parent structure* used by InChI, with canonical atomic numbers.

This model allows one to tune the identifier's resolving power, its "granularity". This is illustrated by Figure 2 (this Figure and related discussion provide just a brief introduction to InChI layers; a more detailed description of the layers can be found later in this paper).

In the InChI string, the core parent structure is encoded by a string composed of several layers. Each layer is a character sequence starting with '/' (forward slash) and followed by a letter denoting the identity of the layer.

Layers represent: empirical formula, the very first layer after the prefix "InChI=1/", in this example “C10H16N5O13P3”; skeletal connections '/c'; hydrogens layer '/h' (indicating positions of immoveable and sharing of moveable Hs); charge layer '/q'; protonation/deprotonation '/p'.

In Figure 2 the parent core structure is derived from the source structure by adding one proton (i.e., one proton must be removed to go back from parent to source, "/p-1"). It has the two groups of skeletal atoms sharing one hydrogen each, "(H,21,22)(H,23,24)", and two other groups sharing two hydrogens each, "(H2,11,12,13)(H2,18,19,20)". Here “H2” denotes two hydrogens and “18, 19, 20” denotes the numbered (non-hydrogen) atoms.

Additional features are represented by the layers appearing further to the right. The stereochemistry layer (“/t4-,6-,7-,10-/m1/s1/” in Figure 2) includes the sublayer for tetrahedral centers '/t' complemented by two indicator stereo layers '/m1' and '/s1'. A double-bond stereochemical layer '/b', may also be present for other structures. [Note that the stereochemical layers may be optional if the stereochemistry is not known or does not need to be specified].

The next layer is the isotopic '/i' layer. Here "/i29 + 1" signifies that atom number 29 consists of the isotope with mass increased by unity with respect to the natural value. Note that the isotopic layer may optionally include its own ‘/s’ stereo sublayer, as adding isotopic substitution to the core parent structure may change the stereogenic elements and their configurations.

The next layer is the "FixedH" '/f' layer, which lists the exact position of tautomeric hydrogens (“/fC10H15N5O13P3/h18,21,23H,11H2/q-1”, Figure 2). Note that specifying the exact position of tautomeric hydrogens may change the ionization pattern considered by the InChI algorithm. Consequently, the FixedH layer may contain its own formula sublayer and charge sublayers (“/fC10H15N5O13P3” and “q-1”). Note also that this layer may optionally include its own ‘/s’ stereo sublayer, as adding exact positions of tautomeric hydrogens to the core parent structure may change the set of stereogenic elements and their configurations.

InChI may be produced not only for a single structure, but also for a combination of components not bound to each other (this may be thought of as representing equimolar mixtures). In InChI, this is termed “disconnected structures.” In this case, each layer includes information about all of the components separated by ';' except for the chemical formula, which is dot-separated. This feature enables InChI to provide representations for metallic complexes, adducts, etc. where the bonding may not be known, ill-defined, or diffuse including the cases where three or more atoms may be involved in a “bond.”

#### Standard and non-standard InChI

The layered structure of InChI allows one to represent a molecular structure with a desired level of detail. Accordingly, InChI software may generate different InChI strings for the same molecule, depending on the choice of a multitude of options (e.g., distinguishing or not distinguishing tautomers). This flexibility, however, may be considered a drawback with respect to standardization/interoperability. In 2009, the ‘standard’ InChI, which is always produced with fixed options, was introduced in response to these concerns.

The standard InChI was defined to ensure interoperability/compatibility between large databases/web searching and to facilitate information exchange. Its layered structure conforms to the following requirements.

Standard InChI distinguishes between chemical substances at the level of ‘connectivity’, ‘stereochemistry’, and ‘isotopic composition’. Connectivity is defined here as tautomer-invariant valence-bond connectivity with different tautomers having the same connectivity/hydrogen layer. The Standard InChI representation for organometallics does not include bonds to the metal. Stereochemistry is defined here as a configuration of stereogenic atoms and bonds where only absolute stereo or no stereo is allowed, and unknown stereo designations are treated as undefined. Isotopic composition is defined here as the mass number of isotopic atoms (when specified).

For InChI version 1, the standard InChI is designated by the prefix “InChI=1S/” (that is, the letter ‘S’ immediately follows the Identifier version number, ‘1’). The non-standard InChI is designated by the prefix: “InChI=1/” (that is, the letter ‘S’ is omitted).

#### InChI valence schema

Additional details of the “InChI model of chemical structure” are concerned with accounting for the specific properties of elements, namely, the *valence schema*.

In particular, in many situations InChI treats metal and non-metal atoms differently. The following elements are considered as non-metals: H, He, B, C, N, O, F, Ne, Si, P, S, Cl, Ar, Ge, As, Se, Br, Kr, Te, I, Xe, At, Rn. All the others are metals.

For all elements, InChI recognizes typical (standard) valence states. These standard valences are summarized in Table 1 (omitting noble gas elements for which valence is zero). Implicit hydrogen atoms are added to hypovalent non-metal atoms in order to reach the nearest higher standard valence, as indicated in Table 1 (however, no addition is made to reach the pentavalent state of neutral nitrogen and the tetravalent state of neutral sulfur atoms). Also, implicit hydrogen atoms are added to the following metal atoms: Li, Be, Na, Mg, Al, K, Ca, Ga, Rb, Sr, In, Sn, Sb, Cs, Ba, Tl, Pb, Bi, Po, Fr, Ra.

**Table 1:** **Standard valences used by InChI**

**Element**	**Atomic charge**				
	**−2**	**−1**	**0**	**1**	**2**
H	-	-	1	-	-
Li	-	-	1	-	-
Be	-	-	2	1	-
B	3	4	3	2	1
C	2	3	4	3	2
N	1	2	3, 5	4	3
O	-	1	2	3, 5	4
F	-	-	1	2	3,5
Na	-	-	1	-	-
Mg	-	-	2	1	-
Al	3, 5	4	3	2	1
Si	2	3, 5	4	3	2
P	1, 3, 5, 7	2, 4, 6	3, 5	4	3
S	-	1, 3, 5, 7	2, 4, 6	3, 5	4
Cl	-	-	1, 3, 5, 7	2, 4, 6	3, 5
K	-	-	1	-	-
Ca	-	-	2	1	-
Sc	-	-	3	-	-
Ti	-	-	3, 4	-	-
V	-	-	2, 3, 4, 5	-	-
Cr	-	-	2, 3, 6	-	-
Mn	-	-	2, 3, 4, 6	-	-
Fe	-	-	2, 3, 4, 6	-	-
Co	-	-	2, 3	-	-
Ni	-	-	2, 3	-	-
Cu	-	-	1, 2	-	-
Zn	-	-	2	-	-
Ga	3, 5	4	3	-	1
Ge	2, 4, 6	3, 5	4	3	-
As	1, 3, 5, 7	2, 4, 6	3, 5	4	3
Se	-	1, 3, 5, 7	2, 4, 6	3, 5	4
Br	-	-	1, 3, 5, 7	2, 4, 6	3, 5
Rb	-	-	1	-	-
Sr	-	-	2	1	-
Y	-	-	3	-	-
Zr	-	-	4	-	-
Nb	-	-	3, 5	-	-
Mo	-	-	3, 4, 5, 6	-	-
Tc	-	-	7	-	-
Ru	-	-	2, 3, 4, 6	-	-
Rh	-	-	2, 3, 4	-	-
Pd	-	-	2, 4	-	-
Ag	-	-	1	-	-
Cd	-	-	2	-	-
In	3, 5	2, 4	3	-	1
Sn	2, 4, 6	3, 5	2, 4	3	-
Sb	1, 3, 5, 7	2, 4, 6	3, 5	2, 4	3
Te	-	1, 3, 5, 7	2, 4, 6	3, 5	2, 4
I	-	-	1, 3, 5, 7	2, 4, 6	3, 5
Cs	-	-	1	-	-
Ba	-	-	2	1	-
La	-	-	3	-	-
Ce	-	-	3, 4	-	-
Pr	-	-	3, 4	-	-
Nd	-	-	3	-	-
Pm	-	-	3	-	-
Sm	-	-	2, 3	-	-
Eu	-	-	2, 3	-	-
Gd	-	-	3	-	-
Tb	-	-	3, 4	-	-
Dy	-	-	3	-	-
Ho	-	-	3	-	-
Er	-	-	3	-	-
Tm	-	-	2, 3	-	-
Yb	-	-	2, 3	-	-
Lu	-	-	3	-	-
Hf	-	-	4	-	-
Ta	-	-	5	-	-
W	-	-	3, 4, 5, 6	-	-
Re	-	-	2, 4, 6, 7	-	-
Os	-	-	2, 3, 4, 6	-	-
Ir	-	-	2, 3, 4, 6	-	-
Pt	-	-	2, 4	-	-
Au	-	-	1, 3	-	-
Hg	-	-	1, 2	-	-
Tl	3, 5	2, 4	1, 3	-	-
Pb	2, 4, 6	3, 5	2, 4	3	-
Bi	1, 3, 5, 7	2, 4, 6	3, 5	2, 4	3
Po	-	1, 3, 5, 7	2, 4, 6	3, 5	2, 4
At	-	-	1, 3, 5, 7	2, 4, 6	3, 5
Fr	-	-	1	-	-
Ra	-	-	2	1	-
Ac	-	-	3	-	-
Th	-	-	3, 4	-	-
Pa	-	-	3, 4, 5	-	-
U	-	-	3, 4, 5, 6	-	-
Np	-	-	3, 4, 5, 6	-	-
Pu	-	-	3, 4, 5, 6	-	-
Am	-	-	3, 4, 5, 6	-	-
Cm	-	-	3	-	-
Bk	-	-	3, 4	-	-
Cf	-	-	3	-	-
Es	-	-	3	-	-
Fm	-	-	3	-	-
Md	-	-	3	-	-
No	-	-	2	-	-
Lr	-	-	3	-	-
Rf	-	-	4	-	-
Db	-	-	5	-	-
Sg	-	-	6	-	-
Bh	-	-	7	-	-
Hs	-	-	1	-	-
Mt	-	-	1	-	-
Ds	-	-	1	-	-
Rg	-	-	1	-	-
Cn	-	-	1	-	-

### Layout of InChI layers

#### Main layer: representing core parent structure

##### Empirical formula sublayer: representing composition

The chemical formula is represented according to Hill convention, that is, beginning with carbon atoms, then hydrogens, then all other elements in alphabetical order. This is the only layer prefixed with a single slash, ’/’, without a following character.

Note that this formula may be different from the one seen for the source chemical structure, as it refers to the core parent structure. If the source structure depicts charged species, the InChI algorithm may protonate or deprotonate it to create a neutral parent (to ensure that the same basic layers will be generated for neutral and ionized forms).

For example, InChI for Cl^−^, “InChI=1S/ClH/h1H/p-1”, has formula sublayer “ClH” (for the proton, InChI has no chemical formula sublayer: “InChI=1S/p + 1”). For the anion of adenosine triphosphate, ATP, InChI has the formula layer "C10H16N5O13P3".

##### Skeletal connections layer

This layer prefixed with ‘/c’ represents connections between skeletal atoms by listing the canonical numbers in the chain of connected atoms (branches are given in parentheses).

Note that the canonical atomic numbers, which are used throughout the InChI, are always given in the formula’s element order. For example, “/C10H16N5O13P3” (the beginning of InChI for adenosine triphosphate) implies that atoms numbered 1–10 are carbons, 11–15 are nitrogens, 16–28 are oxygens, and 29–31 are phosporus. Hydrogen atoms are not explicitly numbered.

##### Hydrogens layer

This layer prefixed with ‘/h’ lists the bonds between the atoms in the structure, partitioned into as many as three sublayers. The first sublayer represents all bonds other than those to non-bridging H-atoms, the second sublayer represents bonds of all immobile H-atoms, and the third sublayer provides locations of any mobile H-atoms. This last sublayer represents H-atoms that can be found at more than one location in a compound due to various types of tautomerism. This sublayer identifies the groups of atoms that share one or more mobile hydrogen atoms. In addition to hydrogen atoms, mobile H groups may contain mobile negative charges. These charges are included in the charge layer.

#### Charge layer

This layer provides information about net charge and is composed of two sublayers.

##### Charge sublayer

This sublayer ‘/q’ is simply the net charge of the core parent structure.

##### Protonation/deprotonation sublayer

This sublayer ‘/p’ indicates the net number of protons removed from or added to the source structure while deriving its core parent.

##### Mesomerism

Mesomerism is the concept related to the situation in which the molecular structure cannot be unambiguously represented by a single classical structural formula; rather, two (or more) mesomeric structures must be drawn and considered to contribute to the overall picture.

As the IUPAC Gold Book states, “mesomerism” is “Essentially synonymous with resonance. The term is particularly associated with the picture of π-electrons as less localized in an actual molecule than in a Lewis formula. The term is intended to imply that the correct representation of a structure is intermediate between two or more Lewis formulae” [28]. In other words, mesomers are considered as imaginary objects (or even drawing artifacts) that cannot be distinguished by a simple chemical identifier.

Mesomerism is effectively eliminated in InChI. Mesomers have the same InChIs (this is true for all possible InChI layouts of layers). Actually, this is very natural. Mesomeric structures of a molecular entity have the same basic connectivity but differ in bond orders, and maybe by having atomic charges on different atoms. InChI does not use bond orders and does not place charges on particular atoms; the placement of hydrogen atoms in a mesomeric system, which would be important for InChI, is always the same.

**Figure 3:**
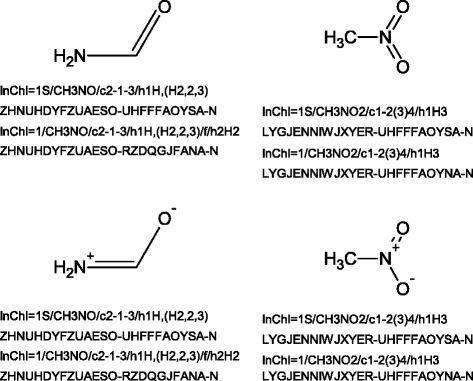
Both Standard (upper 2 lines under each drawing) and FixedH (lower 2 lines) versions of InChI and InChIKey for mesomers are the same: formamide (left) and nitromethane (right).

**Figure 4:**
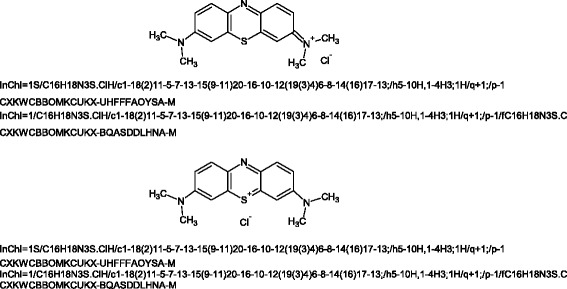
Both Standard (upper 2 lines under each drawing) and FixedH (lower 2 lines) versions of InChI and InChIKey for mesomers are the same, as exemplified by Methylene Blue.

This is illustrated by Figure 3, which shows mesomers of formamide and nitromethane as well as the associated InChIs. A more complex example, Methylene Blue, is presented in Figure 4. Again, all InChIs for the mesomers are the same (note that their Symyx NEMA keys differ, as is shown in [20]).

Note that all the above discussion about mesomerism and mesomers is equally applicable to aromaticity and resonance structures.

#### FixedH layer

This layer prefixed with ‘/f’ serves as the exact specification of tautomers. When potentially mobile H atoms are detected and the user specifies that they should be immobile (tautomerism not allowed), this layer binds these H atoms to the atoms specified in the input structure. In the case where this causes a change in earlier layers, appropriate changes are added to this layer (earlier layers are not affected).

**Figure 5:**
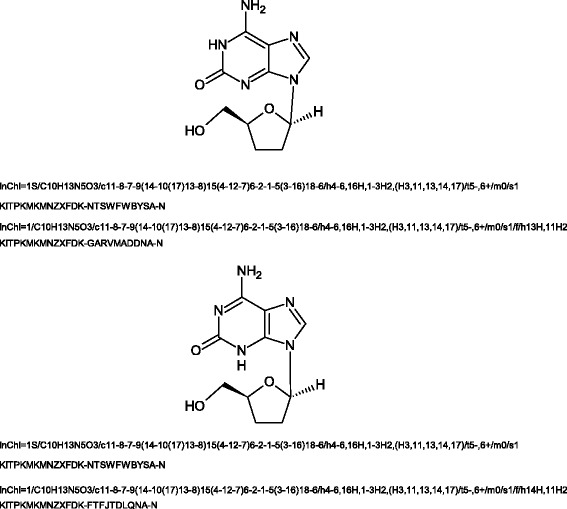
Standard versions (upper 2 lines under each drawing) of InChI and InChIKey for tautomers are the same while FixedH versions (lower 2 lines) differ, as exemplified by an isoguanosine derivative.

Tautomers have the same Standard but different FixedH InChIs (and InChIKeys), Figure 5 shows this in the example of an isoguanosine derivative (this example was also used in [20] where it was noted that the tautomers have different Symyx NEMA keys but the same InChIKeys; however, only Standard InChIKeys were quoted).

#### Stereochemistry layer

##### Overview of stereochemistry layer with its sublayers

The stereochemical layer contains sublayers representing double bond stereochemistry and tetrahedral stereochemistry (including allenes).

The values in this layer depend on the contents of preceding layers. For example, the value produced for the stereo layer will depend on whether it was derived from a main layer or Fixed-H layer or whether it belongs to an isotopic layer. Therefore, this type of layer may be present at several locations in an Identifier.

Two distinct classes of stereochemistry are represented, sp^2^ (double bond or *Z/E*) and sp^3^ (tetrahedral). The double bond sublayer ‘/b’ precedes the tetrahedral sublayer ‘/t’. These sublayers do not affect each other if involved substituents are constitutionally different. Otherwise, the content of each sublayer may influence another one. For example, if the two stereo-enabled (chiral) ligands at the same end of a double bond are constitutionally identical, the double bond stereo depends on tetrahedral stereo configurations of these two ligands.

##### Double bond sp^2^ (Z/E) stereo layer ‘/b’

Expression of a stereo configuration is easily done in two-dimensional drawings.

When double bonds are rigid, stereoisomerism is readily represented without ambiguity. However, in some cases in alternating bond systems, non-rigid bonds may be formally drawn as double bonds.

Bonds in these systems, when discovered by InChI algorithms, are not assigned stereo labels. InChI does not generate sp^2^ stereoisomerism information in small rings (less than 8 atoms).

##### Tetrahedral stereo layer ‘/t’

Tetrahedral (typically, sp^3^) stereochemistry is readily represented using conventional wedge/hatch (out/in) bonds commonly employed in 2-D drawings. Relative tetrahedral stereochemistry is represented first, optionally followed by a tag to indicate absolute stereochemistry.

**Figure 6:**
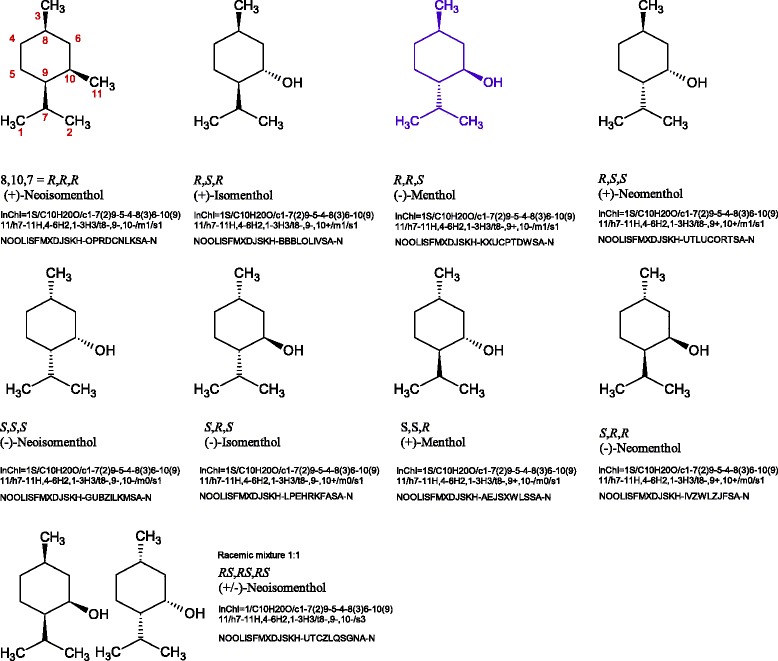
Stereoisomers of menthol with associated InChI/Keys.

In general, the InChI algorithm marks the configuration of a stereogenic center or bond as either ‘+’ or ‘-‘, as shown in Figure 6. These marks have no relation to *R,S* or *E,Z* configurations (actually, they are based on considering canonical atomic numbers of substituents at the stereogenic center). When a stereo center configuration is not known , an ‘unknown’ descriptor may be specified (which will appear in the stereo layer). If a possible stereocenter is found, but no stereo information is provided, it will be represented in a stereolayer by a not-given (‘undefined’) flag.

In current InChI software v. 1.04 (2011) a question mark (‘?’) is used, by default, for both ‘undefined’ and ‘unknown’ flags. However, in a non-standard InChI generated with option ‘SLUUD’ turned On, the symbol ‘u’ is used to indicate explicitly entered ‘unknown’ stereo (while ‘?’ is retained for ‘undefined’).

#### Isotopic layer

The Isotopic layer (signified with the prefix ‘/i’) identifies different isotopically labeled atoms. Exchangeable isotopic hydrogen atoms (deuterium and tritium) are listed separately. The layer also contains any changes in stereochemistry caused by the presence of isotopes.

#### Reconnected layer: coordination compounds and organometallics

To avoid many ambiguities that typically arise when representing metal-containing compounds, the InChI algorithm breaks bonds to metal(s), that is, it “disconnects” these compounds. More details of disconnection procedure may be found in Section “Normalization of input structure”, sub-Section “Breaking bonds to metal atoms”.

The original metal bonding scheme is preserved in so-called “reconnected layer”, which is optionally included into Identifier. This layer is signified with the prefix '/r' and simply contains all the layers appearing in the case where the InChI string is generated without breaking bonds to metal atoms. That is, ‘/r’ is followed by formula and all the other subsequent layers whichever are applicable.

### InChIKey

InChIKey is a compact chemical identifier derived from InChI. The InChIKey is always only 27-characters long. Consequently, it is a much more convenient identifier for searching the internet and indexing databases (see Figure 1). Indeed, based on conversations with search engine developers, it is a practical requirement for the InChIKey to be all upper case letters and a length that all search engines will accept without truncation or modification. A disadvantage of the InChIKey is that one loses the ability to algorithmically restore a structure from a textual label: InChIKey is a *structure-based registry-lookup identifier*, see Background section.

Finding the structure corresponding to a given InChIKey requires searching on the Web or querying dedicated resolvers (e.g. those of ChemSpider [29] and NCI [30]; both are free to use). Of course, for specific targeted databases a lookup service may be added by developers/maintainers (as is implemented, e.g., in the UniChem [31] database Web services [32]).

InChIKey is an encoded version of the hash codes calculated from a source InChI string, elaborated with convenience “flag symbols”.

Hashing is a one-way mathematical transformation typically used to calculate a compact fixed length digital representation of a much longer string of arbitrary length^d^. As the hash function maps input values, strings, to the strongly compacted space, getting the same hash code for two different inputs (*collision*) is unavoidable. Of course, collision means loss of identifier’s uniqueness. However, the use of appropriate hashing details typically allows one to successfully utilize hash codes in various identification tasks.

By design, a goal of InChIKey is to partially preserve the hierarchical layered structure of the parent InChI. The first block of 14 (out of total 27) characters for an InChIKey encodes core molecular constitution, as described by formula, connectivity, hydrogen positions and charge sublayers of the InChI main layer. The other structural features complementing the core data -– namely, exact positions of mobile hydrogens, stereochemical, isotopic and metal ligands, whichever are applicable -- are encoded by the second block of InChIKey. The possible protonation or deprotonation of the core molecular entity (described by the protonation sublayer of the InChI main layer), is encoded in the very last InChIKey flag character, see below.

As a result, the first InChIKey block is always the same for the same molecular skeleton. All isotopic substitutions, changes in stereoconfiguration, tautomeric state and coordination bonding are reflected in the second block.

InChIKey inherits the Standard or non-standard nature of the parent InChI (signified by a dedicated flag character). This inherited nature influences the “resolving power” of the identifier. For example, Standard InChIKey (produced from Standard InChI) does not account for tautomerism. In addition, it may also indicate only absolute stereo. It also does not account for the bonds of the original structures to metal atoms, if they were present and disconnected on Standard InChI generation.

Shown below is the current format of InChIKey (please note that this is different from the initial format, which appeared in 2007, Software version 1.02-beta release).

AAAAAAAAAAAAAA-BBBBBBBBFV-P

All the symbols except the delimiter (a dash that is a minus sign) are uppercase English letters representing a “base-26” encoding. The overall length of InChIKey is fixed at 27 characters, including separators (dashes). As mentioned previously, lower case letters would be useless as web search engines do not differentiate between upper and lower case for searching.

Here are the five distinct components:

1. AAAAAAAAAAAAAA
  The first block: 14-characters encoding core molecular constitution.
2. BBBBBBBB
  The second block: 8-characters encoding advanced structural features whichever are applicable (stereochemistry, isotopic substitution, exact position of mobile hydrogens, metal ligation data).
3. F
  Flag character: either ‘S’ for Standard InChI parent or ‘N’ for non-standard.
4. V
  Version character: currently, ‘A’, which means 1.
5. P
  Protonation/deprotonation flag.

‘N’ means no proton-related ionization (“Neutral”). Other options are:

**Diagram 1:**
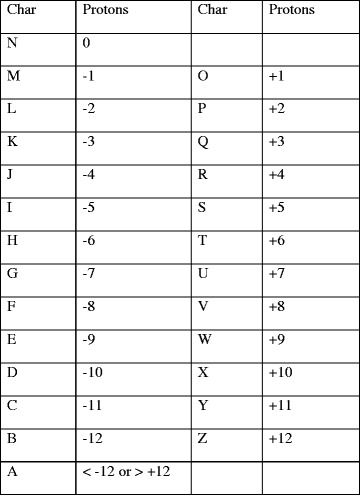
ᅟ

**Figure 7:**
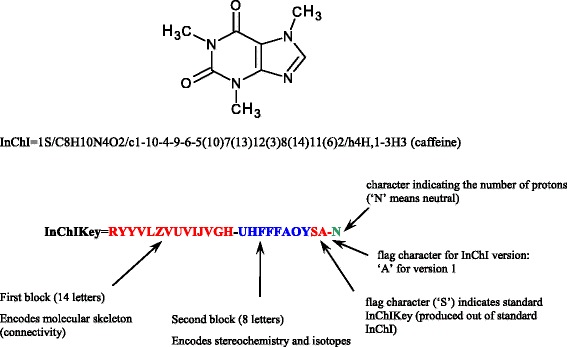
InChIKey layout explained (using caffeine as an example).

This layout is exemplified in Figure 7.

**Figure 8:**
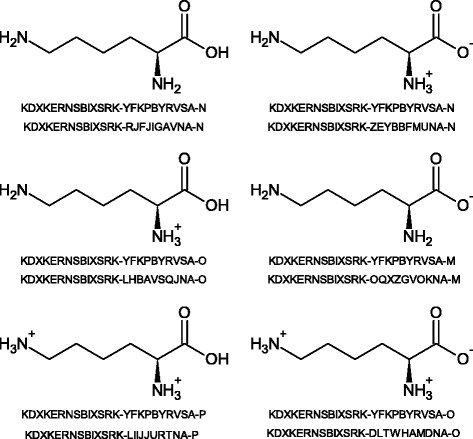
Standard (upper line under each drawing) and FixedH (lower line) InChIKeys for the various ionization states of L-lysine.

Note that different protonation states of the same compound will have Standard InChIKeys that differ only by a single character, the protonation flag (unless both states have number of inserted/removed protons > 12). Moreover, since neutral and zwitterionic states of the same molecule have the same zero number of inserted/removed protons, they will also have the same Standard InChIKeys. However, non-standard InChIKeys generated from non-Standard InChIs (including FixedH sublayer) will allow one to distinguish between the states. This is exemplified by InChIKeys for various ionization states of L-lysine, Figure 8.

## Overview of implementation

### General workflow

**Figure 9:**
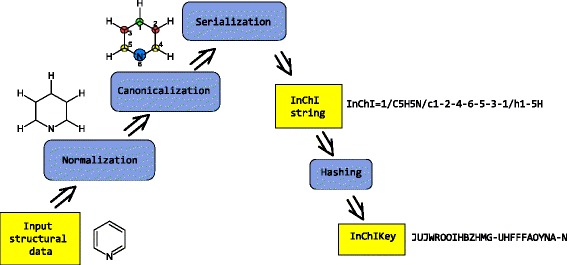
General workflow of InChI/Key generation.

The general workflow of derivation of InChI from structural data is illustrated by Figure 9. There are three major steps in the workflow: (a) normalization of input structure, that is, converting the supplied structural data into internal data structures conforming to the InChI chemical model; (b) canonicalization of atomic numbering, which accounts for atomic equivalence/inequivalence relations appearing under this model; and (c) serialization, that is, generating the final sequence of symbols, an InChI string. There is an optional fourth step (d); hashing of the InChI string and producing a compact InChIKey.

### Input data

The input data for InChI generation is the structure represented in “classical chemical structure” paradigm as atomic and bonding data, with optional addition of “0D” stereochemical data. The data may be supplied either as a molfile or SD file (input of inchi-1 executable, see InChI Software User’s Guide [33]) or C data structures (as described in the header file “inchi_api.h”, see InChI source code).

Each atom is described by a number of properties: its chemical element name; *x,y,z*- coordinates (all or any of them may be zero); list of bonded atoms; either the exact number of implicit hydrogen atoms (with separate indication for protium, deuterium, and tritium, if applicable) or flag signifying that implicit hydrogens should be added; isotopic mass; radical state; formal integer charge.

Each bond is described by its type and stereochemistry indicator, if applicable. A bond type may be single, double, or triple. “Resonant” or “aromatic” is not allowed; a chemical structure described with aromatic bonds should be explicitly converted to a representation with alternating single and double bonds, prior to serving as InChI input. For end user convenience, as aromatic bonds may occur widely in molfiles and SD files (in explicit violation of file format specification [21]), they are typically tolerated by inchi-1 executable, which itself performs a conversion; however, the success is not guaranteed. The stereochemistry indicator is in wedge convention (preferably, in one-wedge style to avoid ambiguity) [34]; it indicates the wedge direction (“up”/”down”/”either”), as well as orientation of the wedge narrow point (towards the atom or out of it). The configurations of stereogenic double bonds are expressed *via* atomic coordinates. If all the coordinates are zero, “0D” stereochemical data may be added to specify configuration (applicable to input for InChI Library, API, procedures).

InChI options are the switches that modify default behavior of InChI algorithms/software; they are described in a separate section.

### Normalization of input structure

The first step of InChI production is normalization: converting the input structural data into data structures conforming to InChI representation rules, organization principles and model of chemical structure.

If applicable, normalization starts from preprocessing, correcting the input structural formula according to several hard-coded “good drawing rules”, intended to ease further treatment. Some of these corrections correspond to mesomeric forms of functional groups as intentionally drawn by chemists (e.g., for nitro groups), while the others serve to correct strange drawing artifacts related to computer origin (which occur surprisingly often in large databases). In particular, normalization serves to exclude the issues concerning alternating bonds, resonance and aromaticity.

The next step of normalization includes breaking bonds to metal ions, as InChI’s core parent structure is always metal-disconnected, to avoid numerous issues with different bonding models for metallated compounds.

The next step is to find protons necessary for dealing with variable protonation, again to ensure elucidation of the (de)protonation-independent core parent structure.

The final normalization step includes the discovery of conventional tautomeric patterns and ‘resonances’ that may occur due to bond alternation or positive charge migration along paths of alternating bonds.

The normalization and the stereochemical perception stages rely heavily on testing whether a bond order can be changed due to the presence of an alternating bond circuit, as well as the possibility of a hydrogen atom, charge, or radical center to migrate along a path of alternating bonds. This testing is based on a matching algorithm described in detail in ref. [35].

For the fixed H layer, only moving positive charges along paths of alternating bonds are allowed.

#### Correcting input structural formula

This includes the following transformations, whichever are applicable to the original structure (note that this step is still performed “within” the classical structure model and extensively operates with bond orders and atomic charges).

##### Moving charge from hydrogen to heavy atom

**Diagram 2:**
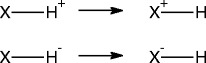
ᅟ

##### Converting charge-separated patterns to neutral

**Diagram 3:**
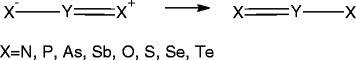
ᅟ

Example:

**Diagram 4:**
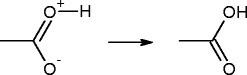
ᅟ

##### Decreasing charge separation by increasing valence

**Diagram 5:**
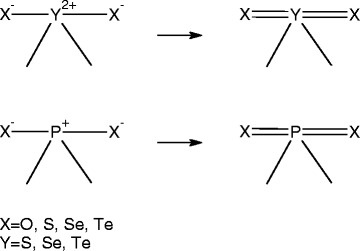
ᅟ

##### Moving negative charge from central atoms in oxoanions

**Diagram 6:**
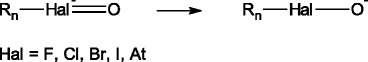
ᅟ

Example:

**Diagram 7:**
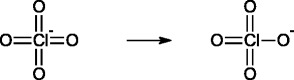
ᅟ

**Diagram 8:**
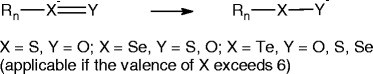
ᅟ

Example:

**Diagram 9:**
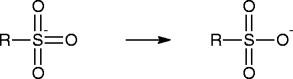
ᅟ

##### Moving positive charge to create imine nitrogen

**Diagram 10:**
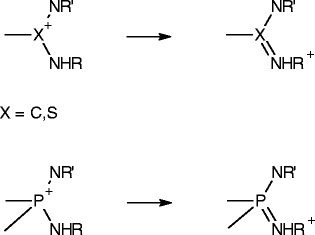
ᅟ

##### Annihilating adjacent opposite charges going to higher valence state

The full set of rules for annihilation of adjacent charges is documented in the InChI Technical Manual [5]. One particularly important rule concerns drawing of nitro and similar groups:

**Diagram 11:**
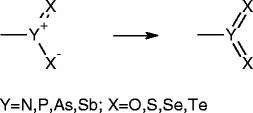
ᅟ

#### Breaking bonds to metal atoms

To avoid many ambiguities that typically arise when representing metal-containing compounds, the InChI algorithm always breaks bonds to metal(s), that is, it disconnects these compounds. However, this is implemented in a different manner for "simple salts" and for coordination/organometallic compounds.

##### Disconnecting simple salts

Simple salts, for InChI, are compounds of type M-X or Y-M-X formed by metal atom M and “acids” HX, HY. Acids here are the substances of the following three kinds:

**Diagram 12:**
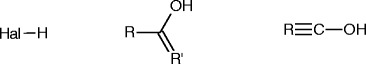
ᅟ

In “salts” drawn connected, metals are connected to the acid by single bonds only and do not have H-atoms connected to them. Metal valences should be the lowest known to InChI valence or, for some metals, the valence may also be the 2nd lowest valence. Positively charged metals should have the lowest valence known to InChI (see Table 1). Upon disconnection, atom X (X = Hal or O) of the acid receives a single negative charge; the charge of the metal is incremented. Substances drawn as H_4_N-X are disconnected to NH_3_ and HX.

Note that compounds formed by many inorganic acids do not fit the above salt definition. For example, sodium nitrate is treated as a coordination compound (so may be reconnected on user request).

Several examples of salt disconnection are shown below:

**Diagram 13:**
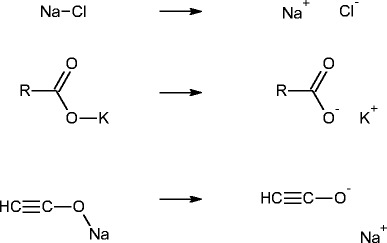
ᅟ

##### Disconnecting other metal-containing compounds

In an effort to deal with the various different conventions used for drawing organometallic compounds, all metal atoms are disconnected in the main layer. In the process, the charges for disconnected halogens, O, S, Se, Te, N, P, As, and B are adjusted if possible by transferring charge to the metal atom.

The InChI algorithm may be instructed, by a software switch, to add to the identifier a “reconnected” layer that contains all bonds given in the input structures, including those to metal. Note that a disconnected “salt” (previous section) cannot be reconnected this way.

At this point rules for annihilating adjacent opposite charges going to higher valence state, see above, are applied a second time, to the disconnected structure.

#### Eliminating radicals and converting aromatic bonds to alternating single and double

This is the first step out of several that may change bonds in the structure in a systematic order along alternating bond paths. Before attempting this change, the algorithm detects bonds (highlighted with red below) and marks them as fixed. The order of these bonds will not be allowed to change.

**Diagram 14:**
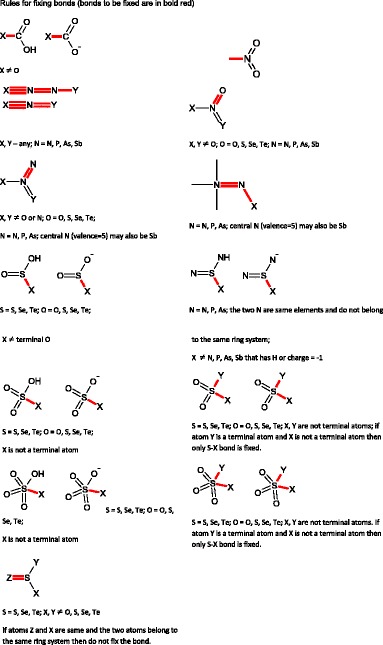
ᅟ

Elimination of radicals can be illustrated as follows:

**Diagram 15:**
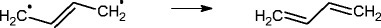
ᅟ

The conversion of aromatic bonds to alternating single and double bonds is done through radical cancellation, for example:

**Diagram 16:**
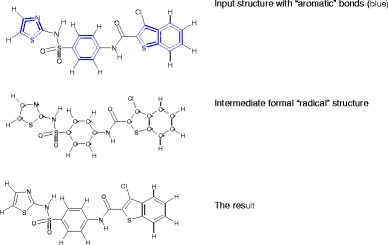
ᅟ

#### Finding [de]protonation pattern which leads to neutral core parent structure

This step occurs for ionized structures. It converts various (de)protonation forms to the same parent neutral structure, memorizing associated changes in the protonation layer.

The necessary condition for this step is a presence, in the input structure, of charges +1 or -1 located on non-metal atoms that have standard valences (see Table 1). The total charge on these atoms is counted and used later. Charges on atoms that are adjacent to other charged atoms are not counted. Non-ring bonds altered during variable protonation processing are marked as non-stereogenic. The so-called aggressive (‘hard’) proton removal or addition procedure is described below.

##### Remove protons from charged heteroatoms

This step removes protons from protonated atoms and places them in a separate proton (charge) layer. If the structure contains atom Y′H_*m*_^+^ (*m* ≥ 1, Y′ is N, P, O, S, Se, or Te), then it is replaced with Y′H_*m*-1_. This is a “simple removal” of a proton. Since some protonated atoms are, in effect, concealed by alternating bond conventions, a separate effort is made to find and disconnect these protons. This “hard removal” involves changing bonds and removing H from formally uncharged atoms. It may be illustrated as follows. If there exist atoms =N^+^ or ≡N^+^ and -NH_*m*_ (*m* ≥ 1, at least one neighbor of N must be Y or Sb) or =Y-QH (Y = C, N, P, As, S, Se, Te, Cl, Br; Q = O, S, Se, Te), then an attempt is made to find a fragment containing an alternating path (a, b,… are other atoms) and remove a proton:

H_*m*_N − b = c − d = N^+^ → H_*m*_N^+^ = b − c = d − N → H_*m*-1_ N = b − c = d − N + H^+^ or

HQ − Y = a − b = c − d = N^+^ < → HQ^+^ = Y − a = b − c = d − N < → Q = Y − a = b − c = d − N < + H^+^

More aggressive transformations are also possible, for example, the following "hard" proton removal:

**Diagram 17:**
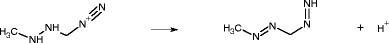
ᅟ

During this process:

- positive charges may be moved between N^+^, N^−^ and N (except N in -N=Q);
- negative charges may be moved between N^+^, N^−^, N, and Q, Z in -Y=Q, =Y=Q, =Y-QX, **≡**Y-QX, -C-ZX, -Q'-QX, **≡**N^+^-QH, =N^+^=Q, -N^−^-QH, where Q is O, S, Se, or Te; Z is S, Se, or Te; X is H or -; Y ≠ C ≠ N may carry ±1 charge; N in -N=Q is excluded; or
- atoms H may be moved between atoms described in (b).

The neutralization of positive and negative charges may occur. A simple exchange of atom H and a negative charge between two atoms without changing bonds is not allowed.

##### Remove protons from neutral heteroatoms

If the total charge is positive and the structure has fragments = C-QH, -Q-QH, C-ZH, or =N-QH, then hydrogen atoms are removed from the fragments and replaced with negative charges until either no more hydrogens are available or the charge has been reduced to zero. This is a “simple removal” of a proton; example:

**Diagram 18:**
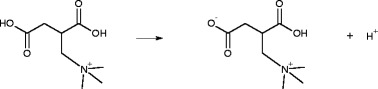
ᅟ

If the total charge is still positive then a “hard proton removal” procedure similar to the previously described one is executed.

During this process:

(d) positive charges may be moved between atoms described in 1 (a);
(e) negative charges may be moved between atoms described in 1 (b);
(f) atoms to receive H if the procedure succeeds: Q in -C=Q, =C=Q, =N^+^=Q, and -N=Q; and
(g) atoms H may be moved between atoms described in 1 (b) except atoms described in (f) above.

If the procedure succeeds, it moves H from atoms described in (g) to atom Q described in (f). After that the H is removed from that Q as a proton, leaving negatively charged O^−^ thus reducing the positive charge.

##### Add protons to reduce negative charge

If the total charge is negative or has become negative due to positive charge removal and the structure has fragments =C-Q^−^, -Q-Q^−^, C-Z^−^, or =N-Q^−^, then protons are added to the fragments replacing negative charges with atoms H until the total charge is reduced to minimal or zero. This is a “simple addition” of a proton.

If the total charge is still negative then a “hard proton addition” procedure similar to the previously described one is executed.

During this process:

(h) positive charges may be moved between atoms described in (a);
(i) atoms to receive negative charge if the procedure succeeds are atoms described in (f):
(j) negative charges may be moved between atoms described in (b) except atoms described in (i) above
(k) atoms H may be moved between atoms described in (b).

If the procedure succeeds it moves negative charge from atoms described in (j) to atom Q described in (i). After that this negative charge is replaced with atom H, which is equivalent to a proton addition thus reducing the negative charge.

#### Analyzing mobile hydrogens and charge

Neutral or singly negatively charged tautomeric atoms and corresponding changeable bonds are detected and marked. Atoms that may exchange hydrogen atoms or negative charges are considered to belong to a “mobile H group”. If positive charges may be moved from an atom as described in the next section, "Moveable positive charge detection", this atom is also considered as possibly tautomeric. Mobile H groups that contain only negative charges are excluded from InChI.

The existence of a ‘protonated’ site is sometimes not readily apparent in a structural drawing. The normalization algorithm is designed to resolve complications that arise from ambiguities introduced at previous step during “hard” or incomplete “simple” removal or addition of protons and in case of charged atoms resembling results of heterolytic dissociation. An example of such ambiguity is shown below:

**Diagram 19:**
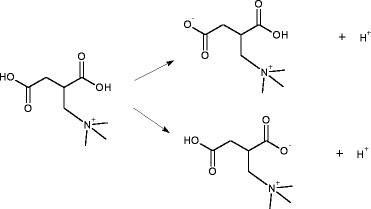
ᅟ

##### Simple tautomerism detection

The main layer must be the same for any arrangement of mobile hydrogen atoms. This is achieved by the logical removal of mobile H-atoms and the tagging of H-donor and H-receptor atoms. To identify these H-atoms we have adopted the straightforward varieties of H-transfer tautomerism listed in Table 2 (see also ref. [36]).

**Diagram 20:**
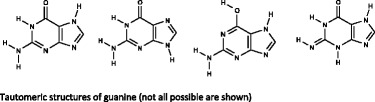
ᅟ

**Diagram 21:**
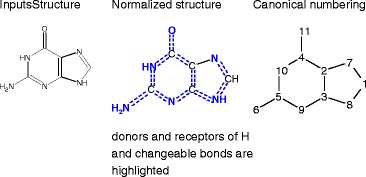
ᅟ

InChI for guanine (optional fixed H layer included) is

**InChI=1/C5H5N5O/c6-5-9-3-2(4(11)10-5)7-1-8-3/h1H,(H4,6,7,8,9,10,11)/f/h8,10H,6H2**

The layers' meaning is:

/h1H**,(H4,6,7,8,9,10,11)**

atom number 1 has one H, 4 atoms H are shared by atoms 6,7,8,9,10, and 11

/f/h**8,10H,6H2**

atom 6 has 2H, atom 8 has 1H, atom 10 has 1H.

**Table 2:** **Tautomerism patterns detected by InChI**

M = Q **−** ZH ↔ MH **−** Q = Z or M = Q **−** Z^**−**^ ↔ M^**−**^ **−**Q = Z			M, Z = N^III^, O^II^, S^II^, Se^II^, Te^II^ (Roman superscripts designate chemical valence)		
			Q = C, N, S, P, Sb, As, Se, Te, Br, Cl, I		
			H = hydrogen, deuterium, or tritium		
The “=” bond may be a double bond, a bond in the alternating single/double bond ring, or a “tautomeric” bond (shown in blue)					
The H atom below can be replaced with a negative charge					
	↔			↔	
	↔			↔	

##### Moveable positive charge detection

Positive charges located on N-atoms are considered moveable along alternating bonds between these atoms. This also applies to phosphorus atoms. Atoms that may exchange positive charges are assigned to a “mobile charge group”. The interference between mobile H and mobile charges may occur.

Hypothetical structures (a), (b), and (c) below serve as an illustration.

**Diagram 22:**
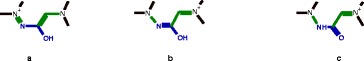
ᅟ

Structure (b) was obtained from structure (a) by formally moving the positive charge from left to right along an alternating bond path. This allows the discovery in structure (b) of a tautomeric pattern (highlighted in **blue**). Bonds that may be changed by moving positive charges are highlighted in **green**. Structure (c) shows another tautomeric form obtained from structure (b). Note that structure (c) does not allow movement of a positive charge back from right to left. These three structures generate the same standard InChI:

InChI=1S/C6H13N3O/c1-8(2)5-6(10)7-9(3)4/h5H,1-4H3/p + 1

but InChI possessing FixedH layer for structure (c) differs from those of structures (a) and (b):

(a,b) InChI = 1/C6H13N3O/c1-8(2)5-6(10)7-9(3)4/h5H,1-4H3/p + 1/fC6H14N3O/h10H/q + 1

(c) InChI = 1/C6H13N3O/c1-8(2)5-6(10)7-9(3)4/h5H,1-4H3/p + 1/fC6H14N3O/h7H/q + 1

For the purpose of detecting stereogenic bonds, the algorithm must also provide a means for testing whether a bond order is changeable. InChI assumes that a changeable bond cannot support *Z/E* stereoisomerism. This is accomplished by introducing fictitious bonds and atoms (used only for internal processing) that represent a mobile H group (red H below) and charge group (red plus below). In the mobile H group fictitious double bonds (red) point to the atom-donors of H or negative charge; in the mobile positive charge group fictitious single bonds point to positively charged atoms.

**Diagram 23:**
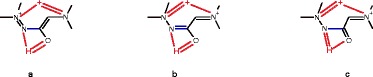
ᅟ

After the discovery of a new mobile group it is added to the structure. This results in the discovery of changeable bonds. In case of the structure (a), adding a charge group allows one to discover changeable bond N-C (shown in blue) and, as a result, discover the mobile H group. These processing steps correct for common ambiguities in input information for conjugated systems where *Z/E* stereochemistry is implied by the drawing, but was not really intended.

##### Additional normalization

As mentioned above, complications arise from ambiguities introduced at “hard” or incomplete “simple” removal or addition of protons and in the case of charged atoms resembling results of heterolytic dissociation. Since there could be more than one possible set of added/removed proton locations or more than one alternating path for “hard” addition or removal, ambiguities may be introduced. These ambiguities are specifically addressed and in most cases fixed (for the details, see InChI Technical Manual [5]).

### Perception of isotopic data

The isotopic structural layer is the most straightforward to compute. In the example below, the isotopic layer is ‘/i1 + 1,4 + 1D’. It contains the canonical atom number followed by the isotopic shift (13 – 12 = +1) followed by isotopic hydrogen (D), if present.

**Diagram 24:**
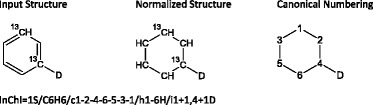
ᅟ

The only complexity arises for isotopically labeled hydrogen atoms that can undergo tautomerism. In the mobile H group these hydrogen atoms are treated as non-isotopic; the number of these mobile isotopic hydrogen atoms is appended to the ”exchangeable isotopic hydrogen atoms” part of the isotopic layer. The same is done to isotopic hydrogen atoms that may be subject to heterolytic bond dissociation in aqueous solution (for example, D in R-SD). Note that it is possible that two isotopic layers appear, one of which is applied to the main layer with mobile H and another to the main layer without mobile H or to the Fixed-H layer, e.g., “InChI=1/CH4N2O/c2-1(3)4/h(H4,2,3,4)/i/hD2/f/h2-3H2/i2D2”.

#### Perception of stereochemical features

The InChI algorithm supports perception of stereochemistry for both 2D (x,y-coordinates of atoms are given; planar depiction) and 3D (x,y,z-coordinates of atoms are given) cases.

For perception of stereo configurations in two-dimensional drawings, the InChI algorithm supports two different systems of wedged and hatched bond interpretations. By default, the convention “narrow end of wedge points to stereocenter”, is used. It suggests that the bond affects the stereochemistry of only one atom. Another - “perspective” - system is invoked by selecting the “narrow end of wedge points to stereocenter is OFF”, “/NEWPSOFF”, option. Here a wedged or hatched bond affects the stereochemistry of the two atoms it connects. Both systems assume that the narrow end of the bond is in the plane of the drawing.

In the 3D-case, the parity is directly calculated from the atomic coordinates, 2-dimensional Up and Down wedged and hatched bond symbols being ignored. However, “Either” (wavy) bonds in the 3-dimensional case still provide “unknown” stereochemistry.

It is straightforward to calculate stereodescriptors in cases where neighbors to a stereogenic element are not constitutionally identical: the parities are calculated from canonical numbers and geometry. Tetrahedral parity is ‘+’ if the canonical numbers of neighbors increase clockwise when observed from a hydrogen atom or an atom that has the smallest canonical number; parity of a double bond is ‘-’ if neighbors with greater canonical numbers are located on the same side of the bond.

When constitutionally identical neighbors are present, several equivalent canonical numberings are possible. To resolve this ambiguity, the algorithm finds a numbering that minimizes a specific internal representation of the stereo layer. In this case, it is desirable to determine whether a possibly stereogenic element is in fact stereogenic. To determine this, the following heuristic approach is used. A pair of constitutionally identical neighbors (termed right and left neighbors) of a possibly stereogenic element is selected. These two neighbors and adjacent atoms are mapped on their constitutionally equivalent counterparts. After the mapping is complete, the canonical numbers are switched between left and right (this leaves the non-stereochemical part of the identifier unchanged). Stereochemical layers corresponding to these two canonical numberings are compared. If the only change that occurs is to the stereogenic element in question and there are not more than two such constitutionally identical stereogenic elements, then these elements are not marked as stereogenic.

##### Double bond stereochemistry

When using input originating from drawings, the perception of formal double bonds capable of supporting *Z/E* isomerism (Table 3) relies on atomic coordinates.

**Table 3:** **Double bonds treated as possibly stereogenic (only one of two atoms connected by a possibly stereogenic double bond is shown)**

	
	
	

In alternating single/double bond cyclic systems, bond-finding algorithms determine whether a formal double bond can exist between each of the two attached atoms. If such a bond can be drawn between sp^2^ hybridized atoms, and the remainder of the π-electron structure can be completed with alternating bonds, that bond is presumed to be a double bond, hence stereogenic (can support *Z/E* isomerism).

Replacement of adjacent charges with incremented bond orders produces structures with two double bonds connected to a nitrogen atom. In reality, one or both double bonds are in place of a single bond or a bond/charge resonance. The rules for stereogenic bond recognition are summarized in Table 4. Recognized stereogenic bonds are drawn in blue.

**Table 4:** **Detection of stereogenic bonds in =N= fragments**

**Input fragment(s)**	**Normalized fragment**	**Interpreted for stereogenic bond detection as**
		

		

		No stereo

		

		No stereo

The InChI supports a ‘not-known’ descriptor for marking double bonds where the *Z/E* isomer is not certain. That is, the stereolayers would be different for (*Z*)-but-2-ene, (*E*)-but-2-ene and but-2-ene.

##### Tetrahedral stereochemistry

Stereochemical descriptors will be processed for tetrahedral atoms such as C, Si and Ge. InChI recognizes the centers listed in Table 5 as capable of supporting sp^3^ stereochemistry.

**Table 5:** **Tetrahedral centers treated as possibly stereogenic**

				
				
				
				

An atom or positive ion **N**, **P**, **As**, **S**, or **Se** is not treated as stereogenic if it has (a) a terminal **H** atom neighbor or (b) at least two terminal neighbors, **−XH**_***m***_ and **− XH**_***n***_, (*n* + *m* > 0) connected by any kind of bond, where **X** is **O, S, Se, Te,** or **N**. Phosphines and arsines are always treated as stereogenic even with H atom neighbors.

The parity of a stereogenic atom is calculated as a volume of an oriented tetrahedron. A wide end of a wedge bond is lifted at an angle of 45° to the plane; a wide end of a hatched bond is lowered at 45° from the plane. Before the volume is calculated all bonds are reduced to the same length. A warning is issued if the central atom is outside of the tetrahedron.

When a complete stereo-description is provided, it is straightforward to derive the InChI for a stereoisomer. Problems may arise for representation of structures that contain inexact stereochemical information. In these cases stereochemical layers of InChI for different input representations of the same substance will match only if they contain precisely the same sets of inexact information. Moreover, stereochemical layers for inexact structures will not match stereochemical layers for a fully described stereoisomer.

Nevertheless, significant interest was expressed for including partial stereochemical information in the InChI. For this purpose, absolute and unknown stereochemical descriptors can be employed, as shown below (left structure is absolute, the C-BrC_2_H stereocenter in the right structure is unknown):

**Diagram 25:**
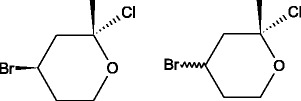
ᅟ

Representing relative and absolute stereochemistry of the whole structure is illustrated for tartaric acid (it is known that the structure is described by either structure 1 or 2):

**Diagram 26:**
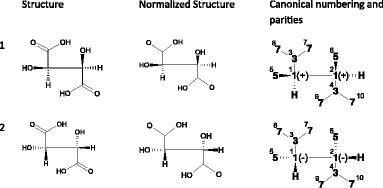
ᅟ

The identifiers for these structures (case of absolute stereochemistry) are

1. **InChI=1S/C4H6O6/c5-1(3(7)8)2(6)4(9)10/h1-2,5-6H,(H,7,8)(H,9,10)/t1-,2-/m1/s1**

2. **InChI=1S/C4H6O6/c5-1(3(7)8)2(6)4(9)10/h1-2,5-6H,(H,7,8)(H,9,10)/t1-,2-/m0/s1**

InChI considers both enantiomers and selects the one that has the “smaller” identifier. /m0 signifies that the selected one has exactly the same stereo arrangement as the input structure; /m1 means that the selected one has the inverse arrangement. /s1 means absolute stereochemistry was requested.

To identify relative stereochemistry the /m segment of the identifier is dropped. As a result the identifiers (case of relative stereochemistry) are the same:

1. **InChI=1/C4H6O6/c5-1(3(7)8)2(6)4(9)10/h1-2,5-6H,(H,7,8)(H,9,10)/t1-,2-/s2**

2. **InChI=1/C4H6O6/c5-1(3(7)8)2(6)4(9)10/h1-2,5-6H,(H,7,8)(H,9,10)/t1-,2-/s2**

/s2 means relative stereochemistry.

The molfile structure format supports the special feature, Chirality Flag. If this flag is set, the tetrahedral stereo is absolute, otherwise relative. The InChI option “Include stereo from chiral flag” (/SUCF command line option) makes InChI calculate tetrahedral stereo according to the Chiral Flag. If Chiral Flag is set, “Include stereo from chiral flag” option is used, and InChI finds that the tetrahedral stereo descriptor does not change upon inversion of the structure, the warning "Not chiral" is issued.

Allenes belong to the tetrahedral layer. However, to indicate stereochemistry of allenes in the input molfile a special effort may be required. Namely, the two bonds at the same end of allene system should be indicated by wedge as stereogenic (and having opposite Up/Down marks). This is a limitation of current InChI software. Cumulenes are treated as double bonds. Table 6 lists the rules used to recognize allenes and cumulenes:

**Table 6:** **Cumulenes treated as possibly stereogenic**

Terminal atoms		
		
Middle atoms		
		

Only cumulenes that have 3 double bonds and allenes that have 2 double bonds are treated as possibly stereogenic. Canonicalization of allene and cumulene stereochemistry is performed together with the double bond stereochemistry.

Some limitations of the InChI algorithm of stereo recognition are considered in the InChI Technical Manual [5].

### Canonicalization

Establishing canonical numbers of chemical graph nodes (atoms) is a problem well-known to chemists, mathematicians and chemoinformaticians and well-discussed in the literature.

For canonicalization that does not involve stereochemistry, the InChI approach is based upon the algorithm by McKay [37,38] (see also explanations in [39]]). This algorithm, modified to accommodate the layered structure of InChI, was implemented in the InChI software.

The stereochemical canonicalization is based on an exhaustive mapping of non-stereochemical canonical numbering on the structure using previously found constitutional equivalence of the atoms. It is an iterative process aimed at establishing the smallest internal representation of the stereochemical layer while keeping other previously found layers unchanged. To avoid combinatorial explosion in the case of highly symmetrical structures, the algorithim uses two approaches: (1) elimination of non-stereogenic elements and (2) a backtrack method that prunes the search tree [40].

**Figure 10:**
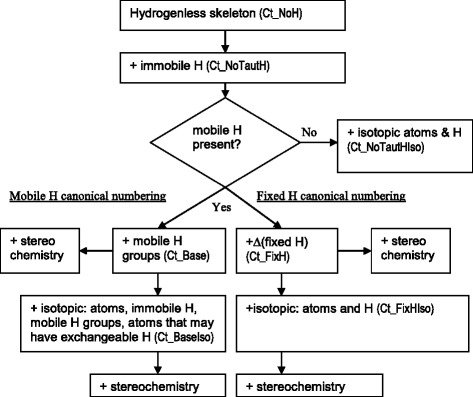
Canonicalization order flowchart.

The canonicalization is performed in stages; each stage adds one more layer to ‘minimize’ while keeping previously found layers unchanged. Figure 10 shows the canonicalization flowchart. As can be seen, the first layer of the Identifier is actually a hydrogenless chemical formula and skeletal connections.

Notes.

- Each set of canonical numberings is a subset of the previous one located up the tree.
- Δ(fixed H) = (number of fixed H on an atom) – (number of H in “mobile-H” structure on the same atom).
- Names in parentheses e.g. (Ct_NoH) are names of data structures in the code.

**Figure 11:**
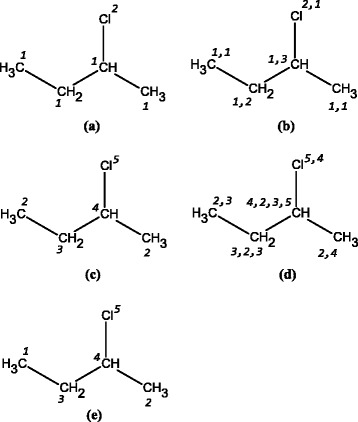
Establishiment of the canonical atomic numbers for 2-chlorobutane (steps **a**-**e** are explained in the text).

Below is a very brief and simplified description^e^ (leaving aside mobile H treatment and technical details; almost all numerical examples below refer to 2-chlorobutane as illustrated by Figure 11).

#### Step A: hydrogenless constitution

1. The skeletal atoms are labelled with numerical "colors" in the following order of precedence.
  - Ordering number of chemical element in the sequence: carbon, other atoms in alphabetic order, bridging hydrogen. In case of C_4_H_9_Cl all C will be given color 1, Cl will be given 2 (Figure 11a).
  - Number of connections (number of bonds). In 2-chlorobutane CH_3_CH_2_CH(Cl)CH_3_ these are (in brackets): C[1]C[2]C[3](Cl[1])C[1]

The resultant "ordered lists of colors" presented in order of the atoms in the semi-structural formula CH_3_CH_2_CH(Cl)CH_3_ are: C: (1, 1); C: (1, 2); C: (1, 3); Cl: (2, 1); C (1, 1) (Figure 11b).

2. Atoms are assigned new colors according to lexicographical comparison of the "color lists", in ascending order [for example, (1,1) < (1,2) < (2,1); (1, 2) < (1, 2, 1)]
  C: 1, 1 = > 2;
  C: 1, 2 = > 3
  C: 1, 3 = > 4
  Cl: 2, 1 = > 5
  C 1, 1 = > 2
  (see Figure 11c)

Notice that each color is equal to the number of atoms that have this or smaller color.

3. Atoms are assigned new "ordered lists of colors": the first in the list is the color of the atom, the rest are sorted in ascending order colors of other atoms, connected to this atom (Figure 11d):
  C: 2, 3
  C: 3, 2, 3
  C: 4, 2, 3, 5
  Cl: 5, 4
  C 2, 4

4. Atoms are assigned new colors according to lexicographical comparison of the "color lists", in ascending order (Figure 11e)
  C: 2, 3 = > 1
  C: 3, 2, 3 = > 3
  C: 4, 2, 3, 5 = > 4
  Cl: 5, 4 = > 5
  C 2, 4 = > 2

5. Steps 3–4 are repeated until all new colors are different or no more changes occur (for 2-chlorobutane the colors - canonical numbers - have already been found, see Figure 11e).

The resultant colors produce a so called equitable partition, in a way which is conceptually almost same as the intermediate result of the SMILES-2 algorithm [17].

6. If some of the colors are still identical, then the smallest is picked up and reduced to the previous color + 1. For example, if colors are (this example does not refer to 2-chlorobutane):

1,2,5,5,5,7,7

then the smallest duplicated color is 5, the previous color is 2. A color of one of the colored-5-atoms will be reduced from 5 to 2 + 1 = 3.

7. Repeat steps 3–6 until all colors become different (this is almost same as obtaining the final result of the SMILES-2 algorithm) and save the "connection table". To make the reading easier, the process of obtaining this table (actually, a list of number) is split into 3 steps.
  - The connection table is made out of segments, ordered in ascending order of the color of the first atom in a segment. The number of the segments is the number of atoms. Each segment starts with the color of an atom and is followed by a colon and a sorted list of the colors of atoms, connected to it:
    1:3; 2:4; 3:1,4; 4:2,3,5; 5:4;
  - Since this connection table contains each connection 2 times (for example, the bond between atoms of color 1 and 3 is in the segments "1:3" and "3:1"), it is rewritten by excluding colors that are greater than the first color in the segment:
    1; 2; 3:1; 4:2,3; 5:4;
  - The delimiters now are redundant because the members of each segment are always smaller than the first member of the segment. This is the final connection table to be saved and used later:
    1, 2, 3, 1, 4, 2, 3, 5, 4

8. There could be a great deal of arbitrariness in choosing the atom whose color was to be reduced at step 6 (in the example, 3 atoms have color 5; each of them could be chosen). Therefore, repeat step 7 for all possible sequences of choosing the atoms whose color is reduced. Lexicographically compare each obtained connection table to the previously saved and keep the smallest one together with the assignment of the colors to the atoms. These colors are the canonical numbers for the hydrogenless structure.

If two connection tables are identical, then atoms that have same colors in two connection tables belong to the same equivalence class; this information is saved and used. The equivalence class is the smallest color in the equivalence group. (This approach may be found in, e.g. [41]. However, the algorithm by McKay implemented in InChI allows one to avoid a combinatorial explosion in typical chemical structures, obtain equivalence classes, and even the order of the permutation group and its generators).

At this time, a canonical numbering (colors) for a hydrogenless structure and the canonical equivalence classes (=the smallest color in each set of equivalent atoms) are obtained.

9. Make new colors out of the canonical equivalence classes and repeat steps 3–8 if these colors are different from the colors previously used at Step 3. Obtain the new minimal connection table. Use these classes as initial colors in the next steps

(If equivalence classes are, for example,

1, 1, 1, 4, 4, 5, 5, 5

then the corresponding colors are

3, 3, 3, 5, 5, 8, 8, 8)

#### Step B. Add hydrogen atoms to the structure

1. Use previously obtained equivalence classes at Step A.9 and use the previously obtained minimal connection table for the comparison. Run Steps A.3-8 with the following difference: each time the connection tables are compared at Step A.8, in case of identical connection tables also compare the list of terminal atoms H in the following form:

$$ 1,\ \mathrm{number}\_\mathrm{of}\_\mathrm{H}(1),\ 2,\ \mathrm{number}\_\mathrm{of}\_\mathrm{H}(2), \dots n,\ \mathrm{number}\_\mathrm{of}\_\mathrm{H}(n) $$

where number_of_H(c) is the number of terminal atoms H attached to the atom that has color c; *n* = number of atoms.

Save the minimal list of the terminal atoms found this way together with the assignment of the colors to the atoms. Also obtain the equivalence classes as was done earlier.

At this time, the canonical colors (numbering) of the non-isotopic non-tautomeric structure are obtained.

#### Step C. Add isotopic composition to the structure

1. If the structure is isotopic, then add one more list to compare whether the connection tables and the lists of terminal atoms H are same:

$$ 1,\ \mathrm{i}\mathrm{s}\mathrm{o}\_\mathrm{weight}(1),\ 2,\ \mathrm{i}\mathrm{s}\mathrm{o}\_\mathrm{weight}(2), \dots n,\ \mathrm{i}\mathrm{s}\mathrm{o}\_\mathrm{weight}\left(\mathrm{n}\right) $$

where iso_weight(c) is the "isotopic weight" of the atom to which the color c was assigned. For each atom the isotopic weight is calculated according to the formula:

$$ \mathrm{i}\mathrm{s}\mathrm{o}\_\mathrm{weight}=n\mathrm{H}1 + 32*\left(n\mathrm{H}2 + 32*\left(n\mathrm{H}3 + 32*\mathrm{shift}\right)\right) $$

where

*n*H1 = number of terminal atoms of protium attached to the atom

*n*H2 = number of terminal atoms of deuterium attached to the atom

*n*H3 = number of terminal atoms of tritium attached to the atom

shift = [(integral) mass of the isotopic atom] - [rounded average atomic mass]

Note: hydrogen H is treated differently from its isotope protium: H has "natural" isotopic composition while protium is treated as an isotopic atom. In case of an atom not isotopic the shift is 0 by definition.

If the atom is isotopic and its mass number is greater than or equal to the rounded average atomic mass (that is, shift is not negative) then the shift is incremented, to avoid shift = 0 for isotopes.

If the formula produces iso_weight equal to 0 (the atom and the attached H are not isotopic) then iso_weight(c) is set equal to a very large number that exceeds any iso_weight. This forces isotopic atoms to assume the least possible canonical numbers.

2. In the case of mobile H the steps are somewhat different, namely:

- (m-a) Add a list of only those H that are not mobile (similar to B.1 above) and minimize both the connection table (it will be same) and the list.
- (m-b) Add mobile groups as pseudoatoms connected by directed edges (it means that these pseudoatoms are not included in the connection table segments of the real atoms) to the atoms where the mobile H and possibly negative charges may reside and canonicalize this structure. Numbers of H and (−) in the groups are in one more list to minimize. The result is the Mobile H group canonical numbering and the corresponding equivalence classes, including equivalence classes of the mobile H (and possibly negative charge) groups. Mobile H groups that have only negative charges are not included in this process.
- (m-c) Add isotopic list (similar to C.1 above) to the number of lists to be minimized. Do not include in it the exchangeable isotopic atoms H. The result of the minimization is the Mobile H canonical numbering and equivalence classes for the isotopic structure.
- (f-a) For the fixed mobile H (FixedH option) start with the results of (m-a) and add a list of the fixed positions of the mobile H (colors of the atoms where these H reside) and numbers of these atoms H. The result of the minimization is the Fixed-H canonical numbering and equivalence classes.
- (f-b) Add isotopic list (similar to C.1 above). The minimization result is the Fixed-H canonical numbering and equivalence classes for the isotopic Fixed-H structure.

Repeat Step B, adding the list of isotopic weights to those already minimized.

At this point the modified algorithm [37] is finished.

It should be pointed out that for the sake of simplicity, avoiding dependence on the hardware or operating system, and the possibility to reproduce the results "by hand", the efficiency of the original McKay algorithm has been reduced. The greatest impact is due to abandoning hashing for the connection table comparison and introducing lists to be minimized additional to the connection table. Also the implemented algorithm for calculating the equitable partition from the given colors is less efficient than the one suggested in ref. [37].

#### Step D. Stereochemistry

1. For the found canonical colors (numbers) calculate double bond >X=Y < and cumulene >W=X=Y=Z < parities. Namely, for each atom at the ends of the double bond or cumulene find connected to it by a single bond the atom that has larger canonical number. If these two found atoms are in "*cis*" positions then the parity is (−), otherwise the parity is (+).

Save parities list *c1*[1], *c2*[1], *p*[1], *c1*[2], *c2*[2], *p*[2],…, *c1*[*n*1], *c2*[*n*1], *p*[*n*1] arranged in ascending order of (c1[i],c2[i]) pairs where *n*1 = number of possibly stereogenic double bonds and cumulenes; *c1*[i] > *c2*[i] are colors of the atoms at the end of a double bond or cumulene; *p*[i] is the parity ("u" > "?" > " + " > "-").

The precedence order is determined as follows. Let a1 > a2 and b1 > b2 be the colors of the atoms for two double bonds, (a1,a2) and (b1,b2). We consider that (a1,a2) > (b1,b2) if and only if (i) a1 > b1 or (ii) a1 = b1 and a2 > b2.

2. For each allene >X=Y=Z < consider a tetrahedron that has as its apices the four atoms connected by single bonds to the allene atoms X and Z. Looking at other apices from the apex that has the smallest canonical number and consider canonical numbers of these three other apices arranged in ascending order. If it is clockwise then the parity is (+), otherwise it is (−).

Save parities list: *c*[1], *p*[1], *c*[2], *p*[2],…, *c*[*n*2], *p*[*n*2] arranged in ascending order of *c*[i], where *n*2 = number of possibly stereogenic allenes; *c*[i] are the colors of atoms Y; *p*[i] are the parities.

3. For each possibly stereogenic atom consider a tetrahedron that has as its apices the four atoms connected this possibly stereogenic atom. If you look at other apices from the apex that has the smallest canonical number and see canonical numbers of these three other apices arranged in ascending order clockwise then the parity is (+), otherwise it is (−).

Save parities list: *c*[1], *p*[1], *c*[2], *p*[2],…, *c*[*n*3], *p*[*n*3] arranged in ascending order of *c*[i], where *n*3 = number of possibly stereogenic atoms; *c*[i] are the colors of the atoms; *p*[i] are the parities.

Note. Terminal hydrogen atoms do not have colors (canonical numbers). In parity calculations, hydrogen atoms are assumed to have colors less than the smallest color of other atoms, that is, less than 1. The values of their colors *c* are assumed to be: *c*[H] < *c*[protium] < *c*[deuterium] < *c*[tritium] < 1. In the special case of all four hydrogen atoms connected to the same atom, the atom is not stereogenic.

In the case of a tetrahedral atom that has only 3 bonds (for example, >S=O or >N-) the direction of the lone electron pair is used as one more bond; *c*[lone pair] < *c*[H].

4. Repeat steps 1–3 for all other mappings of the canonical numbers on the atoms that produce same results as in Step B or C and find the mapping(s) that produce the lexicographically smallest result in this order of the lists: D.1, D.2, D.3.

To each result apply a heuristic to detect possibly stereogenic elements that in reality are not stereogenic; if such elements have been found then remove their parities and repeat D.1-4.

5. Repeat steps 1–4 for the spatially inverted structure. Accept the one that has smaller stereo (D.1 stereo should be same, except cases of constitutionally identical neighbors differing in tetrahedral stereochemistry). Set "inverted" flag if the inverted stereo was selected.

This procedure is applied to all connected components of the whole structure.

### Serialization

The sequence of InChI layers and sublayers is strictly determined; generating the corresponding sequence of characters is mostly a technical issue that does not need specific comments.

It is noteworthy that some complication comes from the fact that InChI may represent a substance composed of several sublayers (connected components) not bound to each other; a definite order of these components must be figured out. For this purpose, the components are sorted (the "greater" component appears first in the InChI string, and descending order of the components is applied) using the comparison function taking into account numerous component data details.

For convenience, the InChI Software may complement an InChI string with an Auxiliary Information (“AuxInfo”) line that provides some explanations of the InChI content and its relation to the input parent structure. In particular, AuxInfo contains mapping of canonical numbers on original atom numbers, information on detected constitutional equivalence of atoms and mobile H groups, stereo of the inverted structure, and reversibility information that is sufficient to recalculate the Identifier and, in case of input from a Molfile, reconstruct the Molfile (except the order of the bonds). The exact AuxInfo layout may be found in the InChI Technical Manual [5].

### Generation of InChIKey

An InChIKey is generated from the corresponding InChI string.

The first step is pre-processing. The very beginning of the source string, either “InChI=1/” or “InChI=1S/” is removed, while the Standard/non-standard nature of the identifier is memorized in the flag character of the InChIKey. The string obtained is split into three parts. Part #1 is the leading substring that comprises formula, connectivity (prefixed by ‘/c’), and hydrogens (‘/h’) sublayers. Part #2 is the protonation layer, prefixed by ‘/p‘. Part #3 is all the rest of the string.

After the pre-processing, hash codes of parts #1 and #3 are calculated separately; then, they are encoded using base-26 schema into the InChIKey first and second blocks. The content of the part #2 is not hashed; instead, it is used to select an appropriate character for the protonation/deprotonation flag.

#### Encoding

The hash code is the sequence of bits. It is represented, in InChIKey, by uppercase English letters (base-26 encoding). This choice is intentional.

Of course, the hash code may be expressed by letters, digits, their mix, or even with bare 0’s and 1’s. However, a particular representation may influence utility for applications like publishing or internet search. Web search engines may tend to break the text "on the border" between letters and non-letters, trying to detect "words" since the words of human languages do not contain digits or punctuation marks. Though the exact behavior may vary, in general, it is more robust to proceed with nothing but letters. Using only letters increases chances that a search engine would consider InChIKey as a single "word" (or phrase) and would index it as such. Also, the robust approach assumes use of only upper-case letters - to avoid possible confusions.

#### Hash codes

InChIKey hash codes are calculated using the SHA-256 cryptographic hash function of the SHA-2 family [42]. Internally, the full 256-bit codes are calculated; then they are truncated to ensure InChIKey’s compactness. (Note that the truncation of the hash code is explicitly allowed by the SHA-2 description).

The hash code going to the first block (representing molecular skeleton, or connectivity) is truncated to 65 bits, and the hash code going to the second block (stereo/protonation/isotopic substitution isomers) – to 37 bits.

A cryptographic hash function is used in order to increase the chances that collision resistance will be as close to the theoretical limit as possible. However, due to the very essence of hash functions, collisions (the same InChIKey for different InChIs/structures) are unavoidable in very large collections.

#### Collision resistance

As was mentioned earlier, a single InChIKey may occasionally map to two or more InChI strings, due to hash codes collision. This is unavoidable for even the perfect hash function, so the only viable approach is to set and keep a level of collision resistance regarded as sufficient for typical applications. At the InChIKey design, this level was placed at the size characteristic of the largest available real-world molecular databases, ≈(50–100) × 10^6^ molecular skeletons; for stereoisomers/isotopomers/tautomers, the practical goal was to avoid collisions up to several thousand isomers for a given skeleton; some margin of safety was also presumed.

Another design goal was to keep InChIKey reasonably short. Balancing and testing determined the current choice of the length of hash codes in the first and the second blocks as 65 and 37 bits, respectively. The estimated level of collision resistance was published when InChIKey was introduced in 2007, and the statement that accompanied this release was: “A theoretical – optimistic – estimate of collision resistance (i.e., the minimal size of a database at which a single collision is expected, that is, an event of the two hashes of two different InChI strings being the same) is 6.1 × 10^9^ molecular skeletons × 3.7 × 10^5^ stereo/isotopomers per skeleton ≈ 2.2 × 10^15^. To exemplify: the probability of a single first block collision in a database of 1 billion compounds is 1.3%. In other words, a single first block collision is expected in 1 out of 100/1.3 = 75 databases of 10^9^ compounds each. For 10^8^ (100 million) compounds in a database this probability is 0.014%.”

Since 2007, two cases of InChIKey collisions have been reported, which prompted us to investigate if the initial estimates are valid. This work is described in a dedicated paper in the *Journal of Cheminformatics* [43] to which the reader is referred for detail. We only quote the conclusion: “the observed statistical characteristics of InChIKey collision resistance are in good agreement with theoretical expectations… the current design and implementation seem to meet their goals”.

### Options available for InChI generation and behavior of InChI algorithms

Note that all switches that modify the InChI act by appending layers, not by altering the core InChI.

#### Structure perception options

These are drawing style/edit flags that affect the input structure interpretation. It is assumed that the user may deliberately use these options to take into account specific features of structure collections. As the result, the perception options may be used for generating Standard InChI without the loss of its "standardness".

The full list of perception options is as follows: DoNotAddH; SNon; NEWPSOFF.

##### DoNotAddH

By default, InChI Software assumes that the input structure may contain "implied" hydrogen atoms and adds hydrogen atoms to eligible atoms to satisfy standard valences. Sometimes, this may produce undesirable results. Option DoNotAddH instructs the software to skip the addition of hydrogen atoms.

##### SNon

This option means that input stereo information (whatever it is and by whatever means it is represented) is completely ignored. That is, InChI generated with SNon option intentionally lacks stereo layer(s). Note that SNon is a "perception option"; therefore, it may be used in the generation of Standard InChI without the loss of its "standardness".

##### NEWPSOFF

By default, when InChI Software analyzes the effect of a wedged bond on the stereo configuration of a tetrahedral stereogenic atom it assumes that the stereo configuration is affected by only those wedged bonds that have the narrow end pointing to the stereogenic atom in question. To use the alternative definition, where a wedged bond affects stereo configurations of both atoms it connects, one may use the option NEWPSOFF ("Narrow End of Wedge Points to Stereo is OFF").

Note that NEWPSOFF is a "perception option"; therefore, it may be used in the generation of Standard InChI without the loss of its "standardness".

#### Stereo interpretation options

These are several options that modify the interpretation of input stereochemical data. In principle, they would be considered related to structure perception. However, as the Standard InChI, by definition, requires the use of absolute stereo (or no stereo at all), these 'stereo interpretation' options assume generation of non-standard InChI.

The stereo interpretation options are: SRel; SRac; SUCF.

SRel assumes that the compound is a single enantiomer but its absolute configuration is not known.

SRac assumes that the compound is a 1:1 mixture of enantiomers.

One more stereo interpretation option SUCF only applies to molfiles in which the CHIRAL flag is set. By default this is set to 0/off. The combinations are:

- SUCF on, CHIRAL 1 = > absolute stereo (default for InChI Software)
- SUCF on, CHIRAL 0 = > relative stereo
- SUCF off, CHIRAL ignored. This defaults to absolute stereo by InChI Software.

Note that many drawing programs do not allow the user to specify the chiral flag so the information is very variable. It is more likely that the maintainer of a collection will know whether some or all of the compounds are of known chirality.

Note that any of the above options makes a non-standard InChI. Even if the compound is not chiral and, as the result, InChI does not have /m and /s segments, any of these options makes the InChI non-standard.

#### InChI creation options

The 'InChI creation' options affect what the InChI algorithm does, not just the structure perception. They modify the defaults specified for Standard InChI and significantly affect the result (e.g., additional InChI layers may appear). Using any of the creation options:

- SUU; SLUUD; RecMet; FixedH; KET; 15T
- makes the resulting identifier Non-standard.
- SUU

By default, InChI Software does not include in the Identifer an unknown/undefined stereocenter unless at least one defined stereo feature is present in the input structure.

The SUU ("always Show Unknown or Undefined stereo") option is intended to alter this behavior. Using SUU results in inclusion of unknown/undefined stereo in all cases.

Note that SUU is an 'InChI creation' option; therefore, it makes a non-standard InChI even if there are no unknown or undefined stereo elements in the structure.

The RecMet option appends the metal-reconnected layer (/r) to the InChI, adding to the identifier the ability to distinguish metal-bonding isomers. This is an 'InChI creation' option; therefore, it makes a non-standard InChI.

The FixedH option appends an additional fixed hydrogen layer (/f) to InChI, adding to the identifier the ability to distinguish tautomers. Note that FixedH is an 'InChI creation' option; therefore, it makes a non-standard InChI.

## Software

The reference implementation of the InChI algorithm is “InChI Software” - the software package distributed (and periodically updated) by the InChI Trust with the approval of IUPAC. The package contains both stand-alone executables as well as the API (Application Programming Interface) Library for InChI generation.

The current InChI Software version 1.04 (the major version of the software is always the version number of the identifier, e.g., 1 for now) includes: inchi-1 - a 'command line' InChI generator, available in 32- and 64-bit versions for MS Windows and Linux; libinchi - InChI API library, available in 32 and 64-bit versions for MS Windows (dll) and Linux (.so library); winchi-1.exe - a graphical Windows application, which provides annotated InChI and AuxInfo together with graphic representation of the original and normalized, canonicalized chemical structure annotated with InChI-related information.

The inchi-1 executable has a normative role i.e. it acts as the final arbiter: by definition, the reference InChI for any molecule is InChI generated with inchi-1.

A distribution package of InChI Software also includes source code for all programs, examples of calling the InChI library, sample molfiles and SDfiles, etc. The source code (all written in pure C) is the ultimate resource for InChI algorithms in maximum detail.

Usage of InChI Software is documented in the User Guide [33] and API [44] (intended for developers who use the InChI library).

InChI Software allows one to produce both Standard and nonstandard InChIs, as well as their hashed representations, InChIKeys. By default, the Standard versions are produced.

Modification of this behavior is achieved through the use of special options ('options' are command-line switches for an executable; they are mirrored by the input parameters of InChI API procedures). If at least one of the options may result in non-standard InChI, the non-standard identifiers are produced.

### Licensing and use of InChI Software

InChI Software is currently distributed under IUPAC/InChI-Trust InChI Licence No. 1.0 [45].

Everybody may read the executable/library code and examine the details of InChI algorithms and their implementation. Everybody as well may freely use the executable or call InChI Software API procedures from within other software.

However, a necessary note is that InChI, by intention, is assumed to have only a single software implementation, the reference implementation provided by IUPAC and InChI Trust (as concerns both the stand-alone executable *inchi-1* and the API library, *libinchi*). Modification of the reference source code is not prohibited, see [45]. However, such modification invalidates the status of the identifier produced by the resulting software as the standard, “IUPAC International Chemical Identifier, InChI”. This means that everybody may modify and use InChI Software source codes in other projects (e.g., for the canonicalization of chemical structures in a non-InChI context). However, no “de novo”/”alternative”/”independent” implementation of InChI is expected.

This approach serves to ensure the standard character of InChI and to avoid a common disaster of conflicting forks/implementations of formally the same “standard”.

## Known problems and limitations

The lack of coverage and limitations for many areas of chemical structures has been noted above. While many of the primary limitations are now being addressed by the various IUPAC Division VIII working groups, some of the most often cited comments are as follows:

- Standard InChI only distinguishes some types of stereo chemistry (e.g., *cis/trans*-platinum structures have the same InChI).
- InChI currently does not handle mixtures well (e.g., stoichiometry, positional isomers, variable bonding situations, polymers).
- InChI is not a file format (the conversion structure - > InChI - > structure can, in a few cases, provide undesirable results).
- InChIKey, the hashed InChI, is limited, in very few cases to date, in terms of variations it can support (i.e., collisions of multiple InChI to one InChIKey).
- InChI does not yet work for large drug molecules (e.g., antibodies with hundreds of amino acids).
- InChI does not handle all tautomers well (1–5, 1–7, 1–9, …, hydrogen shifts, etc.) in standard InChI, which is now being addressed by a new working group.
- Standard InChI does not honor bonds to metals.
- InChI is difficult to read for humans.

## Future prospects

As noted in this manuscript there are many areas of chemistry that need to be and will be addressed. The reader is encouraged to visit the IUPAC [1] and InChI Trust [3] web sites to learn about current and future plans for expansion and extension of InChI.

## Conclusions

InChI is the International Chemical Identifier developed under the auspices of IUPAC with principal contributions from NIST and the InChI Trust. It is a non-proprietary, Open Source, chemical identifier possessing the following principal features:

- structure-based approach;
- strict uniqueness of identifier;
- applicability to the entire domain of “classic organic chemistry” and, to a significant extent, to inorganic compounds;
- ability to generate the same InChI for structures drawn under (reasonably) different styles;
- hierarchical, layered, approach allowing to encode the molecular structure with different levels of “granularity”/different set of layers (a Standard InChI is specifically created for inter-operability);

InChI is complemented by its counterpart compact (hashed, fixed-length) representation, an InChIKey.

To date, InChI and InChIKey were proved to be useful tools for linking various pieces of chemical information.

## Endnotes

^a^As one of the anonymous referees has correctly pointed out, there is a difference between “substance” and “chemical substance”. For short, we use in this paper the term “substance” always to mean “chemical substance”, in the sense of IUPAC Gold Book definition, see below.

^b^The term “InChI” is used in the following text not only for “International Chemical Identifier” itself but also for “InChI algorithms and software” (whence statements like “InChI removes hydrogen…”); the exact meaning is evident from the context.

^c^The result was obtained using InChI Software v. 1.03 and StdInChI; round-trip conversion experiments are documented in InChI v. 1.03 Software Release Notes available on the InChI Trust Download page [6] as a part of v. 1.03 documentation.

^d^The idea of hashing InChI was suggested by Simon Quellen Field at a Google seminar on InChI in 2006.

^e^Please note that the original version of this brief description (previously posted by one of the authors (DT; http://sourceforge.net/p/inchi/mailman/message/1619786/) to the inchi-discuss mailing list and then republished for the wider community by Apodaca in his “Depth-First” blog, http://depth-first.com/articles/2006/08/12/inchi-canonicalization-algorithm/) did contain some minor typos.
